# A Structure–Activity Relationship Study of Trypargine and Opacaline β-Carbolines

**DOI:** 10.3390/md24070248

**Published:** 2026-07-17

**Authors:** Dan Chen, Florent Rouvier, Jean Michel Brunel, Brent R. Copp, Melissa M. Cadelis

**Affiliations:** 1 School of Chemical Sciences, The University of Auckland, Private Bag 92019, Auckland 1142, New Zealand; 2 INSERM, SSA, Aix-Marseille University, MCT, Faculté de Pharmacie, 27 bd Jean Moulin, 13385 Marseille, France

**Keywords:** marine natural products, SAR, opacaline A

## Abstract

The marine environment represents a rich source of novel antimicrobial compounds. This study investigated the isolation and synthesis of antimicrobial agents from the New Zealand ascidian *Pseudodistoma opacum* to identify natural products and derivatives with potential pharmacological applications. Screening of a marine natural product library and related synthetic analogues revealed several compounds exhibiting antibiotic-potentiating activity in combination with doxycycline against *Pseudomonas aeruginosa*. To further explore these findings, the most active natural product, opacaline A (**1**), was synthesized, and a structure–activity relationship (SAR) study was conducted to identify key structural features required for activity. This work reports the first total synthesis of opacaline A (**1**) and racemic 7-bromohomotrypargine (**3**). Biological evaluation demonstrated that the presence of a bromine substituent and a free amine or guanidine group is essential for both intrinsic antimicrobial activity and antibiotic potentiation. Additionally, both oxidation states of the β-carboline ring (aromatic and tetrahydro-) were found to retain activity. These findings provide insight into the structural requirements for activity and support further development of these compounds as antimicrobial adjuvants.

## 1. Introduction

Antibiotic resistance is a persistent global health threat associated with high morbidity and mortality. Bacteria that are resistant to more than one antibiotic are considered multidrug-resistant organisms, and this can occur in all Gram-positive and Gram-negative bacteria. Multidrug resistance can lead to cases where the infections become untreatable with conventional or even last-resort antibiotics [1,2,3,4,5,6]. Gram-negative bacteria, in particular, can be more difficult to treat due to their outer lipopolysaccharide membrane which protects the bacteria from antibiotics which would normally damage the peptidoglycan layer [1,2,3,4,5,6].

Many approaches have been explored to address antimicrobial resistance, including natural product isolation of bioactive materials, antibody therapy, antimicrobial peptides, bacteriophages and microbiota-based therapies [1,2,3,4,5,6,7]. Antibiotic production itself is an ancient evolutionary adaptation, having emerged in microorganisms long before human use of antimicrobial drugs [8]. However, the widespread clinical use of antibiotics has increased selective pressure on bacterial populations, accelerating the spread of resistance through both horizontal gene transfer and the selection of resistant strains. Competition for resources among microorganisms also drives the production of compounds that inhibit competing species [8,9,10,11].

Marine organisms similarly produce a diverse range of secondary metabolites that support survival and defense. Many of these compounds have been identified through natural product isolation, a traditional yet highly effective approach to drug discovery that continues to yield novel biologically active molecules. Marine natural products (MNPs) have been isolated from bacteria, fungi, algae, and marine invertebrates including sponges, corals, cnidarians, arthropods, echinoderms and tunicates. Numerous studies have reported antimicrobial MNPs representing diverse structural classes such as peptides, polyketides, terpenoids, alkaloids, and halogenated compounds [12,13]. Most antimicrobial MNPs have been isolated from marine-derived bacteria, although notable examples have also been identified from sponges, corals, and tunicates.

The ascidian genus *Pseudodistoma*, comprising sessile, filter-feeding marine invertebrates, contains approximately 40 species and has yielded a wide variety of structurally diverse natural products including amino-alcohols (e.g., distaminolyne A, *Pseudodistoma opacum* [14,15]), purines, butanolides (e.g., cadiolide C, *Pseudodistoma antinboja* [16]), β-carbolines and quinones. Among these, distaminolyne A exhibited modest activity against *Escherichia coli* (MIC 64 μg/mL), *S. aureus* (MIC 32 μg/mL) and *Mycobacterium tuberculosis* (MIC 32 μg/mL) [15]. In contrast, cadiolide C exhibited significant antimicrobial activity against *Staphylococcus aureus* ATCC 6538, *Staphylococcus epidermis* ATCC 12228, *Kocuria rhizophila* ATCC 9341 and *Bacillus subtilis* ATCC 6633 at MICs of 0.4 μg/mL, 0.4 μg/mL, 0.3 μg/mL and 3.1 μg/mL, respectively. Additionally, cadiolide C demonstrated strong activity against Methicillin-susceptible *S. aureus* (MSSA) and Methicillin-resistant *S. aureus* (MRSA) with MIC values ranging from <0.13 μg/mL to 0.5 μg/mL [16].

**Figure 1:**
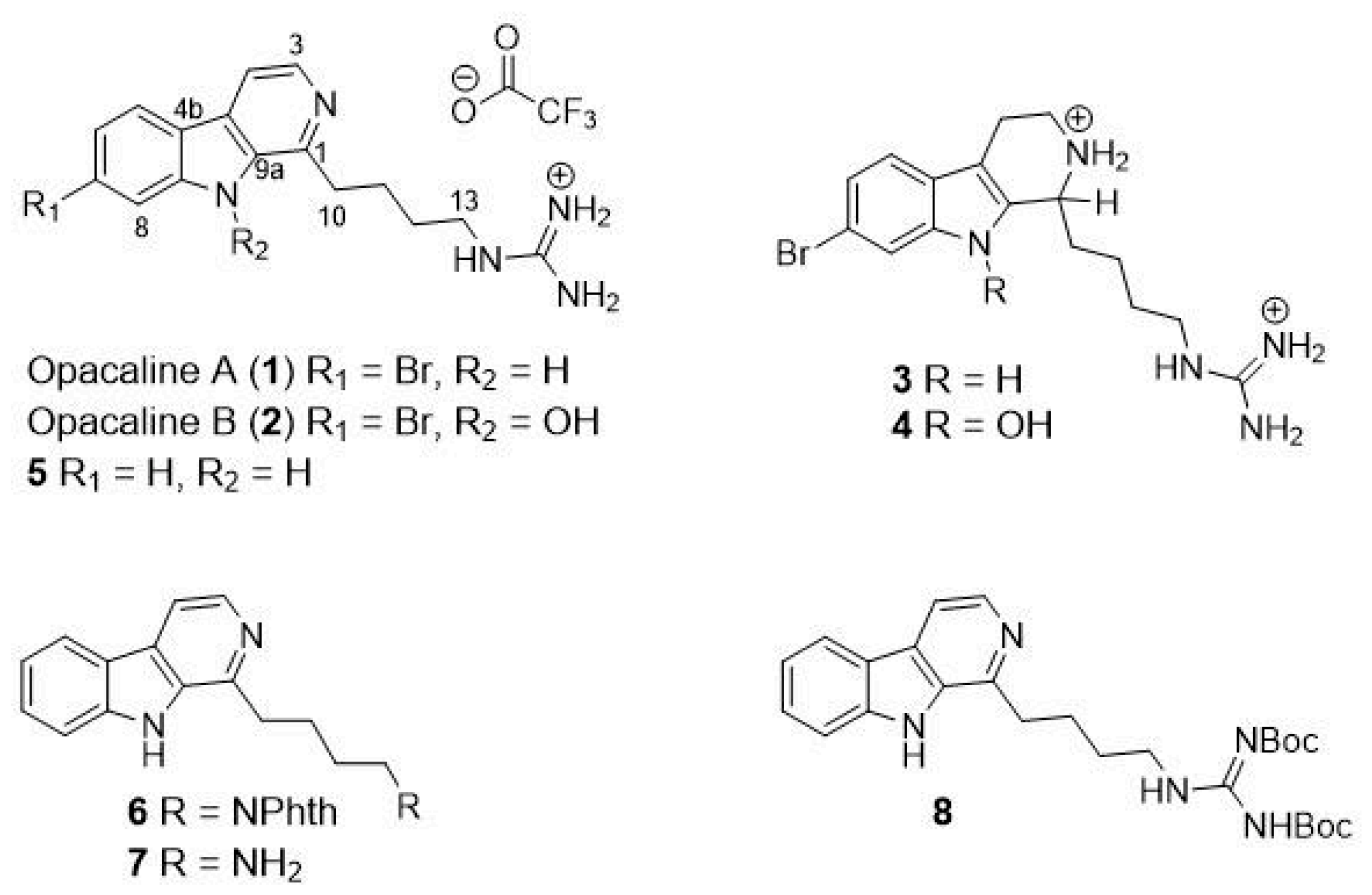
Structures of alkyl-guanidine containing β-carbolines and tetrahydro-variants isolated from *Pseudodistoma opacum* (**1**–**4**) and related des-bromo analogues (**5**–**8**) [15,17].

Motivated by these findings, we screened an in-house library of marine natural products (**1**–**4**) isolated from *Pseudodistoma opacum* and related des-bromo analogues (**5**–**8**) (Figure 1) for intrinsic antimicrobial activity and for their ability to potentiate the effects of doxycycline and erythromycin against *Pseudomonas aeruginosa* and *E. coli*, respectively. Herein, we report preliminary screening data alongside a structure–activity relationship (SAR) study, guided by the results of this screening, investigating the role of halogenation and the importance of the guanidine functional group in mediating both intrinsic antimicrobial effects and antibiotic-potentiating activity.

## 2. Results and Discussion

Preliminary screening of our in-house library of natural products from *Pseudodistoma opacum* (**1**–**4**) and related des-bromo analogues (**5**–**8**) identified opacaline A (**1**) and 7-bromo-*N*-hydroxyhomotrypargine (**4**) (Figure 1) [15,17] as possessing notable antimicrobial activity against *S. aureus*, with MIC values of 6.6 μM and 10 μM, respectively (Table 1). In contrast, opacaline B (**2**) and (−)-7-bromohomotrypargine (**3**) exhibited only moderate activity, with MIC values of 25 μM and 50 μM, respectively.

Compounds **1**–**4** also demonstrated antibiotic-potentiating activity in combination with doxycycline at 2 μg/mL against *P. aeruginosa* and/or erythromycin at 2 μg/mL against *E. coli*. Notably, opacaline B (**2**) showed an eight-fold reduction in MIC against *P. aeruginosa* when co-administered with doxycycline (from 100 μM to 12.5 μM), and a four-fold reduction against *E. coli* with erythromycin (from 100 μM to 25 μM) (Table 2). (−)-7-Bromohomotrypargine (**3**) exhibited an even greater potentiation effect, with a 16-fold decrease in MIC against *P. aeruginosa* (from 800 μM to 50 μM). Similarly, compound **4** displayed an eight-fold reduction in MIC (from 328 μM to 41 μM). The most pronounced activity was observed for opacaline A (**1**), which showed a 32-fold decrease in MIC against *P. aeruginosa* in combination with doxycycline (from 211 μM to 6.59 μM), and a 16-fold reduction against *E. coli* with erythromycin (from 105 μM to 6.59 μM).

Among the des-bromo analogues, the fully aromatic β-carboline **5** displayed only moderate potentiating activity with doxycycline against *P. aeruginosa* (from 506 μM to 126 μM) and no detectable activity against *E. coli*. In contrast, amine **7** exhibited weak potentiating effects in assays with both doxycycline against *P. aeruginosa* and erythromycin against *E. coli*.

To identify the structural features responsible for these activities, a total synthesis and structure–activity relationship (SAR) study was undertaken. The study examined brominated and non-brominated analogues, as well as guanidinylated and non-guanidinylated derivatives, followed by evaluation of their biological activities.

### 2.1. Chemistry

**Scheme 1:**
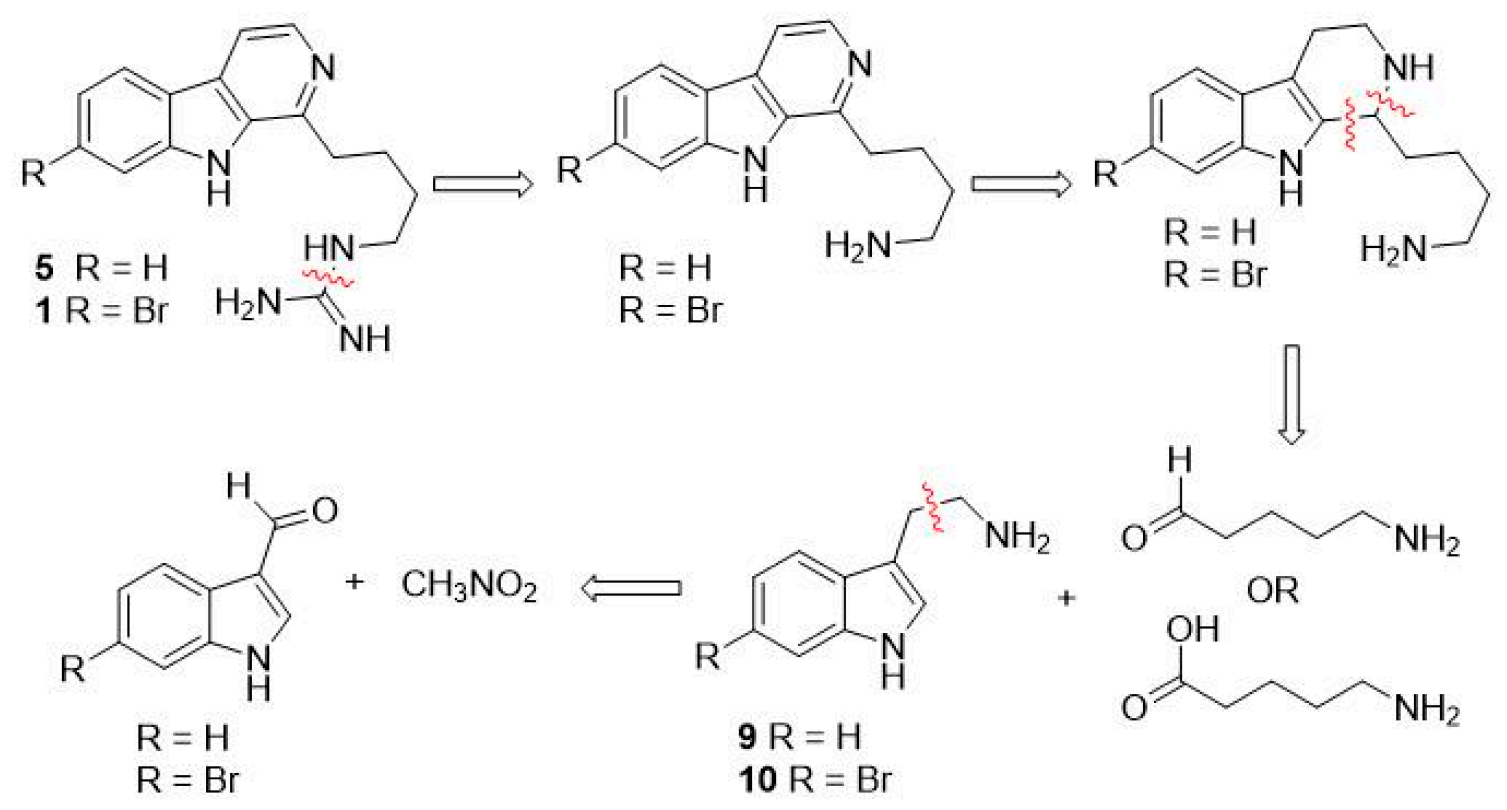
Retrosynthetic analysis of opacaline natural products.

Synthesis towards the β-carboline natural products, and analogues, was accomplished by a Pictet-Spengler (PS) cyclisation. The retrosynthesis is depicted in Scheme 1 showing β-carboline formation by oxidisation of the tetrahydro-β-carboline intermediate and subsequent guanidinylation. The tetrahydro-β-carboline precursor is formed from a Pictet-Spengler (PS) cyclisation of tryptamine (**9**)/6-bromotryptamine (**10**) with the corresponding amino aldehyde.

**Scheme 2:**
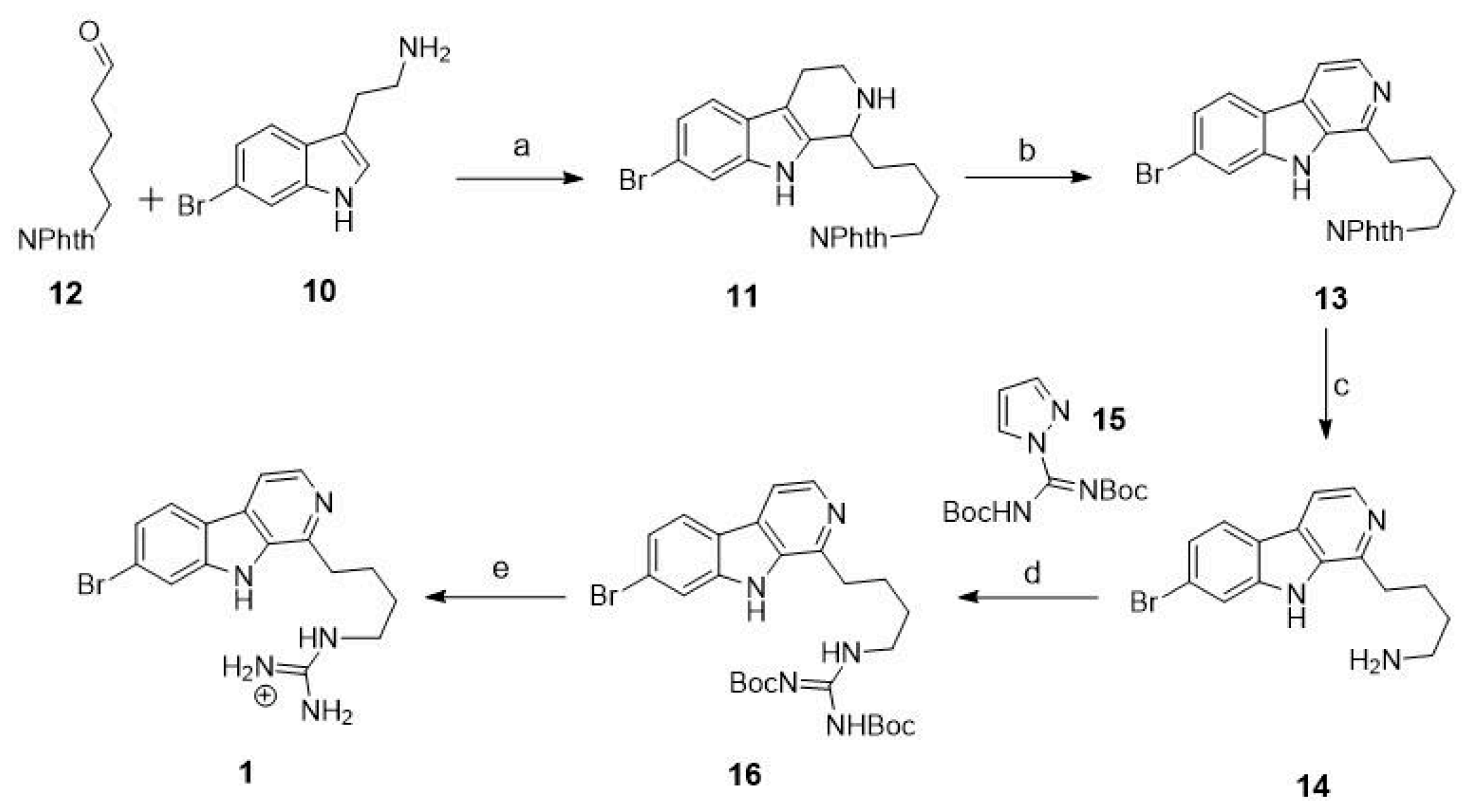
Synthesis of opacaline A (**1**). Reagents and conditions: (a) TFA, microwave 100 °C, 10 min; (b) DDQ, CH_2_Cl_2_, 40 °C, 7 min; (c) hydrazine monohydrate, EtOH, r.t., N_2_, 2–3 days; (d) *Bis*-Boc pyrazole carboxamidine (**15**), DIPEA, DMF, r.t., N_2_, 24 h; (e) 10% TFA, CH_2_Cl_2_, r.t., 2–4 h.

The formation of the PS cyclized tetrahydro-β-carbolines intermediate **11** was achieved under microwave conditions (Scheme 2). Aldehyde **12** [18] was reacted with 6-bromotryptamine (**10**) [19] at 100 °C for 10 min with trifluoroacetic acid (TFA) as the catalyst in a yield of 42%. Analysis of the ^1^H NMR spectrum for **11** identified the presence of an α-proton (H-1) and diastereotopic signals corresponding to H_2_-3a and H_2_-3b and H_2_-10a and H_2_-10b, as well as the absence of an aromatic signal from the indole ring, confirming the formation of the tetrahydro-β-carboline intermediate **11**. Oxidation of the β-carboline ring of **11** was achieved with DDQ in CH_2_Cl_2_ at 40 °C, but this reaction gave a very low yield with **13** obtained in 11% yield (Scheme 2). Other oxidizing reagents were trialed such as KMnO_4_ and Pd/C with microwave conditions, however no significant improvement in yield was observed compared to DDQ.

Cleavage of the phthalimide group utilized hydrazine monohydrate in EtOH to produce amine **14** in 25% yield. Subsequent guanidinylation with *bis*-Boc pyrazole-carboxamidine (**15**) in the presence of DIPEA afforded **16** in 55% yield. Boc deprotection under standard conditions using 10% TFA in CH_2_Cl_2_ for 2–4 h afforded opacaline A (**1**).

**Scheme 3:**
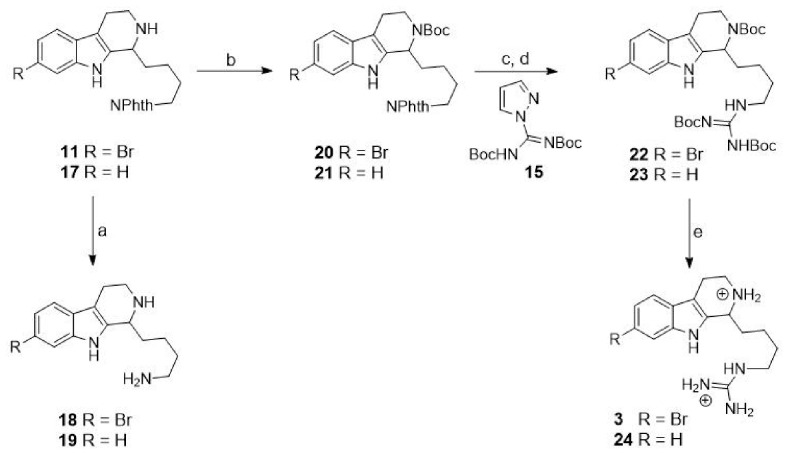
Synthetic route to (±)-7-bromohomotrypargine (**3**) and (±)-homotrypargine (**24**). Reagents and conditions: (a) Hydrazine monohydrate, EtOH, r.t., N_2_, 2–3 days; (b) (Boc)_2_O, Et_3_N, MeOH, r.t., N_2_, 18 h; (c) hydrazine monohydrate, EtOH, r.t., N_2_, 2–3 days; (d) *Bis*-Boc pyrazole carboxamidine (**15**), DIPEA, DMF, r.t., N_2_, 24 h; (e) 10% TFA, CH_2_Cl_2_, r.t, 2–4 h.

For the synthesis of tetrahydro-β-carbolines, intermediates **11** and **17** [17], were deprotected using hydrazine monohydrate in EtOH to afford amines **18** and **19**. Unexpectedly, guanylation did not occur with the free amines **18** and **19**. It was speculated that this was due to the secondary nitrogen atom in the piperidine ring interfering with the reaction. Therefore, the NH group of intermediates **11** and **17** was Boc protected (Scheme 3), forming **20** and **21**, which then allowed the selective deprotection of phthalimide group in the next step to form the free amines which were then subjected to guanidinylation with *bis*-Boc pyrazole carboxamidine (**15**), affording the tri-Boc protected compounds **22** and **23** in 29% and 66% yield over 2 steps, respectively. Global deprotection with 10% TFA in CH_2_Cl_2_ for 2–4 h afforded (±)-7-bromohomotrypargine (**3**) and (±)-homotrypargine (**24**) in 78% and 67% yields, respectively.

A total of three target β-carboline–containing compounds were synthesized, including the first reported total syntheses of the natural products opacaline A (**1**) and racemic (±)-7-bromohomotrypargine (**3**). The overall yield for the synthesis of opacaline A (**1**) was 1.2%, whereas the tetrahydro-β-carboline **3** was obtained in a higher yield of 7.2%. The major limitation in the synthetic route was the DDQ-mediated oxidation step used to generate intermediate **13**, which exhibited the lowest yield. In contrast, the syntheses of (±)-7-bromohomotrypargine (**3**) and (±)-homotrypargine (**24**) did not require oxidation of the piperidine ring and therefore avoided this significant loss in overall yield.

### 2.2. Antimicrobial Activity

*In vitro* assays to assess the antimicrobial properties of the synthesized β-carbolines and tetrahydro-β-carbolines were conducted against a selection of bacterial pathogens *P. aeruginosa* PAO1, *E. coli* ATCC 25922 and *S. aureus* ATCC 25923 (Protocol S1) and the results are summarized in Table 3. Antibiotic-potentiating activity was also evaluated for all these compounds with doxycycline at 2 μg/mL against *P. aeruginosa* and with erythromycin at 2 μg/mL against *E. coli* (Protocol S2), the results of which are presented in Table 4.

The antimicrobial activity of synthetic opacaline A (**1**) was comparable to that of the natural product in preliminary screening assays. In contrast, most synthetic analogues exhibited little or no antimicrobial activity. Notable exceptions were the phthalimide-protected brominated tetrahydro-β-carboline **11**, which inhibited *S. aureus* with an MIC of 44 μM, and the synthetic (±)-7-bromohomotrypargine (**3**), which exhibited an MIC of 84 μM. The reduced potency of synthetic tetrahydro-β-carboline **3** compared with the natural product may be attributable to its racemic nature, whereas the natural product was optically active.

More interesting results were obtained with the antibiotic-potentiating assays (Table 4). The results from the potentiation assays indicated that most synthetic opacaline derivatives that had protected nitrogen groups were inactive and the compounds that possessed terminal amine groups or deprotected guanidine groups exhibited some activity. The free amine compounds **14** and **18** possessed moderate antibiotic-enhancing activity in combination with doxycycline at 2 μg/mL against *P. aeruginosa* with a four-fold decrease in MIC for **14** and an eight-fold decrease in MIC for **18**. In the potentiating assays with erythromycin at 2 μg/mL against *E. coli*, all three compounds **14**, **18** and **19** showed antibiotic-enhancing activity exhibiting a four-fold decrease in MIC. Once again, synthetic opacaline A (**1**) retained the potentiation activity of the natural product, exhibiting comparable potency.

Among the synthetic guanidine-containing compounds (**1**, synthetic **3** and **24**), tetrahydro-β-carboline **24** was inactive in all antibiotic potentiation assays. In contrast, (±)-7-bromohomotrypargine (**3**) enhanced the activity of both antibiotics, reducing the MIC from >338 μM to 42 μM (8-fold) in combination with doxycycline against *P. aeruginosa* and from >338 μM to 21 μM (16-fold) in combination with erythromycin against *E. coli*. Notably, the potentiation of erythromycin against *E. coli* exceeded that of the natural product, (−)-7-bromohomotrypargine. 

The most active compound was still opacaline A (**1**) which exhibited a 32-fold decrease in MIC value in combination with doxycycline against *P. aeruginosa*, and a 16-fold MIC decrease with erythromycin against *E. coli.* Cytotoxicity, tested against human embryonic kidney cells (HEK293, Sigma Aldrich, Bayswater, VIC, Australia) (Protocol S3) and hemolysis assays, tested against human red blood cells (RBC, HEK293, Sigma Aldrich, Bayswater, VIC, Australia) (Protocol S4), showed that opacaline A (**1**) was also non-cytotoxic (IC_50_ > 32 µg/mL) and non-hemolytic (HC_10_ > 32 µg/mL).

Overall, it could be seen that the β-carboline compounds with protected amine groups were less likely to possess antimicrobial or antibiotic-potentiating activity, and the free amine and guanidine-containing compounds were more likely to possess activity. In particular, the bromine-containing β-carboline derivatives were more active compared to the non-brominated versions of the same compound. In addition, the compounds that possessed guanidine groups exhibited more potent antibiotic-potentiating activity compared to the compounds that only possessed a terminal amine group. Between the active compounds of the free amines **7**, **14**, **18** and **19**, the tetrahydro-compound **18** possessed slightly better potentiating activity compared to its fully aromatic version, **14**, while it was the opposite for the guanidine-containing compounds, where the fully aromatic **1** was more active than its tetrahydro-variant, **3**.

**Figure 2:**
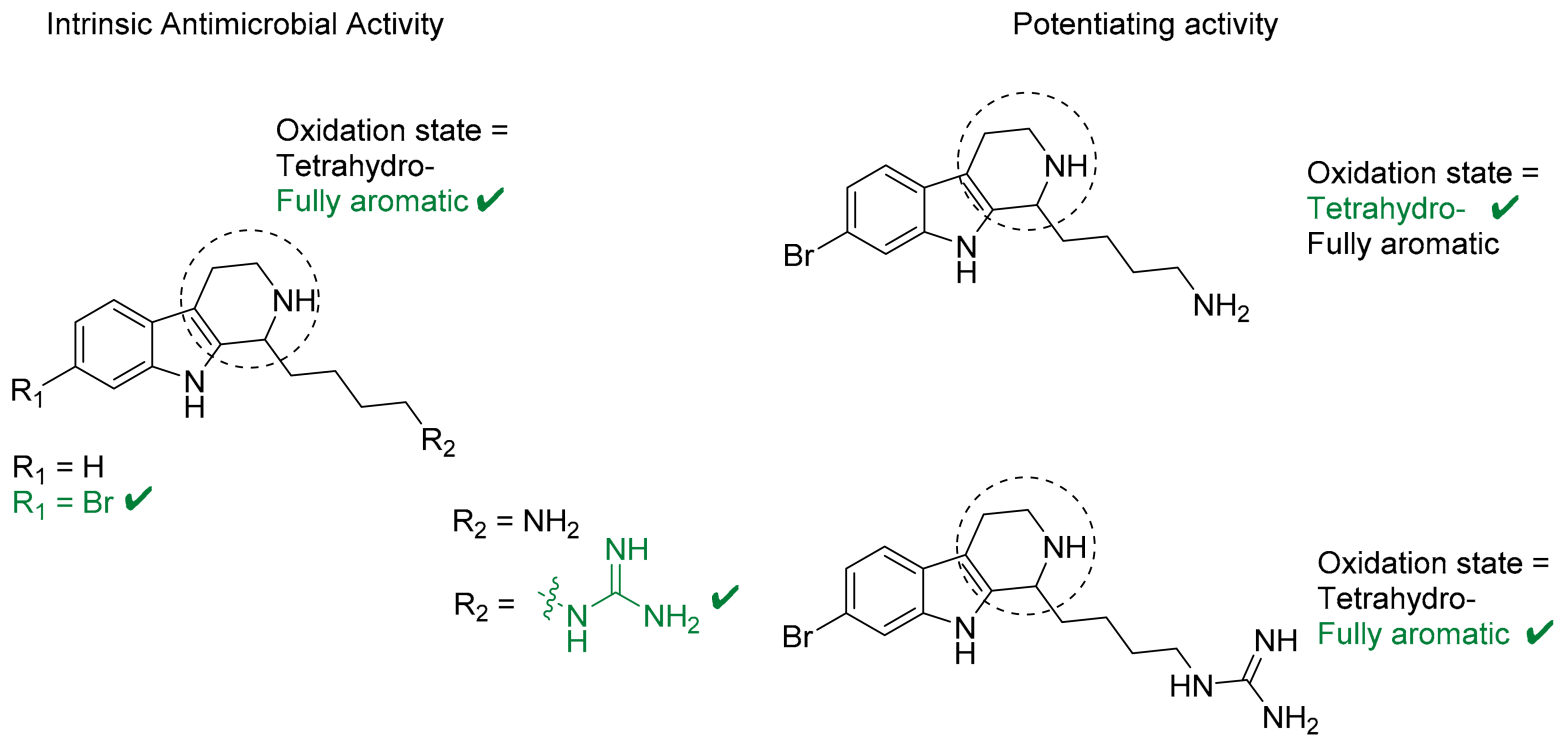
Summary of the necessary components for biological activity for the synthetic β-carboline compounds.

In conclusion, three target β-carboline-containing compounds, inspired by preliminary antimicrobial screening data of β-carboline natural products, were synthesized via a Pictet–Spengler reaction. As part of the SAR investigation, the target final compounds and a series of intermediates were evaluated for antimicrobial and antibiotic potentiating activity. The biological results indicate that it is necessary to possess a bromine group in the β-carboline for better activity. The guanidine-containing compounds are more active as antibiotic enhancers compared to their truncated amine versions; however, when it comes to the oxidation state of the β-carboline ring, the guanidine-containing compounds with a fully aromatic β-carboline structure are more favourable for potentiating activity (Figure 2, bottom right) and intrinsic antimicrobial activity (Figure 2, left), but the truncated amine version favours the tetrahydro-variant for potentiating activity (Figure 2, top right).

## 3. Materials and Methods

### 3.1. General Methods

Mass spectra were recorded using a MicrOTOF-QII mass spectrometer (Bruker Daltonics, Bremen, Germany) coupled with a KD Scientific syringe pump or using a HRMS Thermo Orbitrap Exploris 120 spectrometer. Infrared spectra were recorded on a Perkin Elmer Spectrum 100 Fourier Transform infrared spectrometer (Waltham, MA, USA) equipped with a universal ATR accessory. Melting points were determined on a Reichert–Hofler block and are uncorrected. All NMR spectra were recorded using a Bruker Avance 400 spectrometer (Karlsruhe, Germany) operating at 400.13 for ^1^H nuclei and 100.62 for ^13^C nuclei. Chemical shifts are expressed in parts per million (ppm) relative to the solvent peaks (CDCl_3_: ^1^H TMS, ^13^C 77.16 ppm; CD_3_OD: ^1^H 3.31, ^13^C 49.0 ppm). Assignments are based on 1- and 2-dimensional NMR experiments and analogue comparisons. Standard Bruker pulse sequences were utilized. Pressurized (flash) column chromatography was carried out with Davisil silica gel (40–60 μm) (Grale Scientific, Ringwood, VIC, Australia) or Merck silica gel (15–40 μm) (Darmstadt, Germany). C_8_ or C_2_ reversed-phase flash column chromatography was carried out using LiChroPrep RP-8 (40–63 μm) (Merck, Darmstadt, Germany) or C_2_ (50 μm, 60 Å) (LukNova, Mansfield, MA, USA). Analytical thin layer chromatography (TLC) was carried out on 0.2 mm thick plates of DC-plastikfolien Kieselgel 60 F_254_ or on DC Kieselgel 60 RP-_18_ F_254S_ plates (Merck, Darmstadt, Germany). All solvents were of analytical grade or better and/or purified according to standard procedures. Chemical reagents used were purchased from standard chemical suppliers and used as purchased. Reactions were heated by using DrySyn^TM^ MULTI (Asynt, Cambridge, UK) inserts while the temperature was monitored using a temperature probe placed into a designated place on the insert. Microwave reactions were conducted in a Biotage microwave machine (Biotage, Uppsala, Sweden) with 2–5 mL reaction vials. Compounds 6-bromotryptamine (**10**) [19], phthalimide-protected aldehyde **12** [18] and 1,2,3,4-tetrahydro-β-carboline **17** [17] were synthesized using literature methods [17,18,19]. Compounds **1**–**8**, for which we report herein new biological activities, were available from our compound collection [15,17].

#### 3.1.1. General Procedure A: Phthalimide Deprotection

To a solution of the phthalimide-protected compound (1 equiv.) in EtOH, hydrazine hydrate (12–15 equiv.) was added and the solution was stirred at r.t. for 2–3 days. The solvent was then removed *in vacuo* and purification was achieved using C_8_ reversed-phase column chromatography (10–20% MeOH:H_2_O).

#### 3.1.2. General Procedure B: Reaction with *N*,*N*′-Di-Boc-1*H*-pyrazole-1-carboxamidine

To the amine-containing compound (1 equiv.) in anhydrous DMF (1 mL), *N*,*N*′-di-Boc-1*H*-pyrazole-1-carboxamidine (1.1 equiv.) and DIPEA (5 equiv.) were added. The reaction mixture was stirred for 24 h at r.t. under N_2_ before the solvent was removed *in vacuo*. Product purification was achieved using silica gel column chromatography (CH_2_Cl_2_–1% MeOH:CH_2_Cl_2_).

#### 3.1.3. General Procedure C: Boc Deprotection

To the Boc-protected compound in CH_2_Cl_2_ (2 mL), TFA (0.2 mL) was added. The reaction mixture was stirred for 2–4 h at r.t. and then the solvent was removed *in vacuo.* Product purification was achieved using C_8_ or C_2_ reversed-phase column chromatography (MeOH:H_2_O (0.05% TFA)).

### 3.2. Synthesis of Compounds

#### 3.2.1. 2-(4-(7-Bromo-2,3,4,9-tetrahydro-1*H*-pyrido [3,4-*b*]indol-1-yl)butyl)isoindoline-1,3-dione (**11**)

To a solution of 6-bromotryptamine (**10**) (100 mg, 0.418 mmol) in dry CH_2_Cl_2_ (3 mL), phthalimide-protected aldehyde **12** (97 mg, 0.419 mmol) and TFA (0.064 mL, 0.84 mmol) were added. The reaction mixture was heated under microwave conditions at 100 °C for 10 min and then the solvent was removed *in vacuo*. The product was purified using silica gel column chromatography (2–3% MeOH:CH_2_Cl_2_) to afford **11** as a yellow gum (80 mg, 42%). R*_f_* = 0.52 (MeOH:CH_2_Cl_2_, 1:9); IR (ATR) *v*_max_ 3198, 2945, 2860, 1703, 1670, 1398, 1183 cm^−1^; ^1^H NMR (CDCl_3_, 400 MHz) δ 8.34 (1H, br s, NH-9), 7.87–7.82 (2H, m, H-17), 7.76–7.70 (2H, m, H-18), 7.52 (1H, d, *J* = 1.7 Hz, H-8), 7.29 (1H, d, *J* = 8.4 Hz, H-5), 7.15 (1H, dd, *J* = 8.4, 1.7 Hz, H-6), 4.12 (1H, br t, *J* = 5.4 Hz, H-1), 3.78–3.68 (2H, m, H_2_-13), 3.34 (1H, dt, *J* = 12.7, 4.6 Hz, H-3a), 3.07–3.00 (1H, m, H-3b), 2.74–2.67 (2H, m, H_2_-4), 1.98–1.84 (2H, m, H_2_-10), 1.84–1.65 (2H, m, H_2_-12), 1.55–1.37 (2H, m, H_2_-11); ^13^C NMR (CDCl_3_, 100 MHz) δ 169.0 (C-15), 136.7 (C-8a), 136.5 (C-9a), 134.2 (C-18), 132.1 (C-16), 126.5 (C-4b), 123.4 (C-17), 122.5 (C-6), 119.3 (C-5), 114.8 (C-7), 114.0 (C-8), 109.3 (C-4a), 52.6 (C-1), 42.7 (C-3), 37.1 (C-13), 33.4 (C-10), 28.2 (C-12), 22.5 (C-4), 22.0 (C-11); (+)-HRESIMS *m*/*z* 452.0966 [M + H]^+^ (calcd for C_23_H_23_^79^BrN_3_O_2_, 452.0968), 454.0964 (calcd for C_23_H_23_^81^BrN_3_O_2_, 454.0951).

#### 3.2.2. 2-(4-(7-Bromo-9*H*-pyrido[3,4-*b*]indol-1-yl)butyl)isoindoline-1,3-dione (**13**)

To tetrahydro-β-carboline **11** (106 mg, 0.23 mmol) in anhydrous CH_2_Cl_2_ (20 mL), DDQ (906 mg, 4.00 mmol) was added. The reaction mixture was stirred at 40 °C for 7 min and then washed with 1M KOH (50 mL) until the aqueous layer was slightly yellow. The organic layers were collected, dried with MgSO_4_ and the solvent removed *in vacuo.* Purification was achieved by silica gel column chromatography (3% MeOH:CH_2_Cl_2_) to afford **13** as a light brown oil (12 mg, 11%). R*_f_* = 0.53 (MeOH:CH_2_Cl_2_, 1:9); IR (ATR) *v*_max_ 3053, 2932, 1703, 1396 cm^−1^; ^1^H NMR (CDCl_3_, 400 MHz) δ 9.76 (1H, br s, NH-9), 8.36 (1H, d, *J* = 5.4 Hz, H-3), 7.95 (1H, d, *J* = 8.5 Hz, H-5), 7.91–7.87 (2H, m, H-17), 7.86 (1H, d, *J* = 1.5 Hz, H-8), 7.79 (H, d, *J* = 5.4 Hz, H-4), 7.76–7.74 (2H, m, H-18), 7.40 (1H, dd, *J* = 8.5, 1.5 Hz, H-6), 3.95 (2H, t, *J* = 6.0 Hz, H_2_-13), 3.31 (2H, t, *J* = 8.2 Hz, H_2_-10), 1.95–1.87 (2H, m, H_2_-12), 1.84–1.77 (2H, m, H_2_-11); ^13^C NMR (CDCl_3_, 100 MHz) δ 169.5 (C-15), 145.8 (C-1), 141.4 (C-8a), 139.1 (C-3), 134.5 (C-18), 134.4 (C-9a), 132.0 (C-16), 128.3 (C-4a), 123.5 (C-17), 123.3 (C-6), 123.0 (C-5), 121.9 (C-4b), 121.0 (C-7), 114.9 (C-8), 113.0 (C-4), 36.5 (C-13), 33.4 (C-10), 28.4 (C-12), 25.5 (C-11); (+)-HRESIMS *m*/*z* 448.0655 [M + H]^+^ (calcd for C_23_H_19_^79^BrN_3_O_2_, 448.0655), 450.0632 (calcd for C_23_H_19_^81^BrN_3_O_2_, 450.0635).

#### 3.2.3. 4-(7-Bromo-9*H*-pyrido[3,4-*b*]indol-1-yl)butan-1-amine (**14**)

Following general procedure A, phthalimide-protected compound **13** (12.6 mg, 0.028 mmol) in EtOH (3 mL) was reacted with hydrazine hydrate (0.020 mL, 0.41 mmol) to afford **14** as a yellow oil (2.2 mg, 25%). R*_f_* = 0.74 (RP-_18_, 10% HCl:MeOH, 1:9); IR (ATR) *v*_max_ 3398, 3094, 2979, 1675, 1201, 1133 cm^−1^; ^1^H NMR (CD_3_OD, 400 MHz) δ 8.47 (1H, d, *J* = 6.3 Hz, H-4), 8.38 (1H, d, *J* = 6.3 Hz, H-3), 8.28 (1H, d, *J* = 8.4 Hz, H-5), 7.94 (1H, d, *J* = 1.5 Hz, H-8), 7.57 (1H, dd, *J* = 8.4, 1.5 Hz, H-6), 3.45 (2H, t, *J* = 7.8 Hz, H_2_-10), 3.03 (2H, t, *J* = 7.7 Hz, H_2_-13), 2.02 (2H, tt, *J* = 7.8, 7.7 Hz, H_2_-11), 1.85 (2H, tt, *J* = 7.8, 7.7 Hz, H_2_-12); ^13^C NMR (CD_3_OD, 100 MHz) δ 145.6 (C-8a), 143.1 (C-1), 135.5 (C-9a), 134.2 (C-4a), 131.5 (C-3), 126.7 (C-4b), 126.3 (C-6), 125.5 (C-5), 120.6 (C-7), 116.7 (C-4/C-8), 116.6 (C-4/C-8), 40.1 (C-13), 30.9 (C-10), 28.1 (C-12), 26.6 (C-11); (+)-HRESIMS *m*/*z* 318.0601 [M + H]^+^ (calcd for C_15_H_17_^79^BrN_3_, 318.0600), 320.0578 (calcd for C_15_H_17_^81^BrN_3_, 320.0580).

#### 3.2.4. *tert*-Butyl (4-(9*H*-Pyrido[3,4-*b*]indol-1-yl)butylamino) (*tert*-Butoxy-carbonylamino) 7-Bromo-methylenecarbamate (**16**)

Following general procedure B **14** (17 mg, 0.053 mmol), *N,N′*-di-Boc-1*H*-pyrazole-1-carboxamidine (18 mg, 0.058 mmol) and DIPEA (0.050 mL, 0.32 mmol) in DMF (1 mL) were reacted to afford **16** as a pale-yellow oil/gum (16 mg, 54%). R*_f_* = 0.63 (MeOH:CH_2_Cl_2_, 1:9); IR (ATR) *v*_max_ 3325, 3144, 2978, 1721, 1620, 1417, 1133 cm^−1^; ^1^H NMR (CDCl_3_, 400 MHz) δ 11.59 (1H, br s, NH-14/NH-16), 10.30 (1H, br s, NH-14/NH-16), 8.43–8.40 (1H, m, NH-9), 8.40 (1H, d, *J* = 5.3 Hz, H-3), 7.96 (1H, d, *J* = 8.4 Hz, H-5), 7.78–7.76 (2H, m, H-4, H-8), 7.36 (1H, dd, *J* = 8.4, 1.5 Hz, H-6), 3.46 (2H, dt, *J* = 6.8, 6.4 Hz, H_2_-13), 3.24 (2H, t, *J* = 7.2 Hz, H_2_-10), 2.03 (2H, tt, *J* = 7.2, 6.6 Hz, H_2_-11), 1.53–1.48 (2H, m, H_2_-12), 1.51 (18H, br s, Boc); ^13^C NMR (CDCl_3_, 100 MHz) δ 163.6 (Boc), 156.9 (C-15), 153.4 (Boc), 145.6 (C-1), 141.3 (C-8a), 139.2 (C-3), 135.3 (C-9a), 128.1 (C-4a), 123.0 (C-5/C-6), 122.9 (C-5/C-6), 121.6 (C-4b), 120.9 (C-7), 115.4 (C-8), 112.6 (C-4), 83.6 (Boc), 80.1 (Boc), 39.1 (C-13), 31.6 (C-10), 28.5 (Boc), 28.2 (Boc), 27.2 (C-12), 24.4 (C-11); (+)-HRESIMS *m*/*z* 560.1866 [M + H]^+^ (calcd for C_26_H_35_^79^BrN_5_O_4_, 560.1867), 562.1844 (calcd for C_26_H_35_^81^BrN_5_O_4_, 562.1847).

#### 3.2.5. 2,2,2 Trifluoroacetate Salt 1-(4-(7-Bromo-9*H*-pyrido[3,4-*b*]indol-1-yl)butyl)guanidine (2,2,2 Trifluoroacetate) (**1**) (Opacaline A)

Following general procedure C, Boc-protected compound **16** (11.4 mg, 0.020 mmol) was reacted with TFA/CH_2_Cl_2_ for 4 h followed by workup and chromatography to afford the TFA salt **1** as a pale-yellow oil (6.25 mg, 65%). ^1^H NMR (CD_3_OD, 400 MHz) δ 8.53 (1H, d, *J* = 6.3 Hz, H-4), 8.38 (1H, d, *J* = 6.3 Hz, H-3), 8.32 (1H, d, *J* = 8.7 Hz, H-5), 7.96 (1H, d, *J* = 1.7 Hz, H-8), 7.59 (1H, dd, *J* = 8.7, 1.7 Hz, H-6), 3.45 (2H, t, *J* = 7.8 Hz, H_2_-10), 3.25 (2H, t, *J* = 7.1 Hz, H_2_-13), 2.04–1.96 (2H, m, H_2_-11), 1.80–1.72 (2H, m, H_2_-12); ^13^C NMR (CD_3_OD, 100 MHz) δ 158.7 (C-15), 145.6 (C-8a), 143.4 (C-1), 135.5 (C-9a), 134.3 (C-4a), 131.4 (C-3), 126.8 (C-7), 126.4 (C-6), 125.6 (C-5), 120.7 (C-4b), 116.7 (C-4), 116.65 (C-8), 42.0 (C-13), 31.2 (C-10), 29.6 (C-12), 26.9 (C-11); (+)-HRESIMS *m*/*z* 360.0819 [M + H]^+^ (calcd for C_16_H_19_^79^BrN_5_, 360.0818), 362.0797 (calcd for C_16_H_19_^81^BrN_5_, 362.0798). NMR data agreed with those reported in the literature [17].

#### 3.2.6. 4-(7-Bromo-2,3,4,9-tetrahydro-1*H*-pyrido[3,4-*b*]indol-1-yl)butan-1-amine (**18**)

Following general procedure A, phthalimide-protected compound **11** (66 mg, 0.15 mmol) in EtOH (5 mL) was reacted with hydrazine hydrate (0.11 mL, 2.2 mmol), which after workup and chromatography, afforded **18** as a yellow oil (18 mg, 37%). R*_f_* = 0.77 (RP-_18_, 10% HCl:MeOH, 1:9); IR (ATR) *v*_max_ 3417, 3229, 2962, 1666, 1434, 1183, 1131 cm^−1^; ^1^H NMR (CD_3_OD, 400 MHz) δ 7.53 (1H, d, *J* = 1.6 Hz, H-8), 7.40 (1H, d, *J* = 8.4 Hz, H-5), 7.18 (1H, dd, *J* = 8.4, 1.6 Hz, H-6), 4.72 (1H, dd, *J* = 8.5, 4.4 Hz, H-1), 3.74 (1H, dt, *J* = 12.5, 4.8 Hz, H-3a), 3.50–3.43 (1H, m, H-3b), 3.12–3.03 (2H, m, H_2_-4), 2.99 (2H, t, *J* = 7.8 Hz, H_2_-13), 2.33–2.24 (1H, m, H-10a), 2.07–1.97 (1H, m, H-10b), 1.86–1.74 (2H, m, H_2_-12), 1.72–1.63 (2H, m, H_2_-11); ^13^C NMR (CD_3_OD, 100 MHz) δ 139.1 (C-8a), 130.9 (C-9a), 126.4 (C-4b), 123.8 (C-6), 120.6 (C-5), 116.9 (C-7), 115.3 (C-8), 107.8 (C-4a), 54.4 (C-1), 42.8 (C-3), 40.3 (C-13), 32.6 (C-10), 28.3 (C-12), 23.2 (C-11) 19.3 (C-4); (+)-HRESIMS *m*/*z* 322.0913 [M + H]^+^ (calcd for C_15_H_21_^79^BrN_3_, 322.0913), 324.0893 (calcd for C_15_H_21_^81^BrN_3_, 324.0893).

#### 3.2.7. 4-(2,3,4,9-Tetrahydro-1*H*-pyrido[3,4-*b*]indol-1-yl)butan-1-amine (**19**) (Nazlinin)

Following general procedure A, 2-(4-(2,3,4,9-tetrahydro-1*H*-pyrido[3,4-*b*]indol-1-yl)butyl)isoindoline-1,3-dione **17** [17] (52 mg, 0.14 mmol) in EtOH (5 mL) was reacted with hydrazine hydrate (0.100 mL, 2.0 mmol) which after workup and chromatography, afforded **19** as a yellow oil (8.3 mg, 24%). R*_f_* = 0.83 (RP-_18_, 10% HCl:MeOH, 1:9); IR (ATR) *v*_max_ 3404, 3262, 2990, 1675, 1201 cm^−1^; ^1^H NMR (CD_3_OD, 400 MHz) δ 7.48 (1H, d, *J* = 7.8 Hz, H-5), 7.37 (1H, d, *J* = 8.1 Hz, H-8), 7.15 (1H, ddd, *J* = 8.1, 1.0, 1.0 Hz, H-7), 7.06 (1H, ddd, *J* = 7.8, 1.0, 1.0 Hz, H-6), 4.73 (1H, dd, *J* = 8.4, 4.3 Hz, H-1), 3.74 (1H, dt, *J* = 12.5, 4.8 Hz, H-3a), 3.50–3.43 (1H, m, H-3b), 3.16–3.03 (2H, m, H_2_-4), 2.99 (2H, t, *J* = 7.7 Hz, H_2_-13), 2.35–2.26 (1H, m, H-10a), 2.08–1.98 (1H, m, H-10b), 1.85–1.74 (2H, m, H_2_-12), 1.71–1.63 (2H, m, H_2_-11); ^13^C NMR (CD_3_OD, 100 MHz) δ 138.3 (C-8a), 129.9 (C-9a), 127.4 (C-4b), 123.5 (C-7), 120.6 (C-6), 119.1 (C-5), 112.4 (C-8), 107.4 (C-4a), 54.6 (C-1), 42.9 (C-3), 40.3 (C-13), 32.7 (C-10), 28.3 (C-12), 23.2 (C-11), 19.5 (C-4); (+)-HRESIMS *m*/*z* 244.1808 [M + H]^+^ (calcd for C_15_H_22_N_3_, 244.1808).

#### 3.2.8. *tert*-Butyl 7-Bromo-1-(4-(1,3-dioxoisoindolin-2-yl)butyl)-1,3,4,9-tetrahydro-2*H*-pyrido[3,4-*b*]indole-2-carboxylate (**20**)

To a solution of **11** (129 mg, 0.28 mmol) in MeOH (5 mL) was added (Boc)_2_O (69 mg, 0.32 mmol) and Et_3_N (0.120 mL, 0.86 mmol). The reaction mixture was stirred for 18 h at r.t. under N_2_ and then the solvent was removed *in vacuo*. Product purification was achieved using silica gel column chromatography (100% CH_2_Cl_2_ –> 1% MeOH:CH_2_Cl_2_) to afford **20** as a beige gum (121 mg, 78%). R*_f_* = 0.90 (MeOH:CH_2_Cl_2_, 1:9); IR (ATR) *v*_max_ 3324, 2974, 2935, 1708, 1397, 1166 cm^−1^; ^1^H NMR (CDCl_3_, 400 MHz) δ 7.87–7.80 (2H, m, H-17/H-18), 7.74–7.69 (2H, m, H-17/H-18), 7.50 (1H, d, *J* = 1.5 Hz, H-8), 7.29 (1H, d, *J* = 8.4 Hz, H-5), 7.16 (1H, dd, *J* = 8.4, 1.5 Hz, H-6), 5.31–5.06 (1H, m, H-1), 4.55–4.22 (1H, m, H-3a), 3.81–3.62 (2H, m, H_2_-13), 3.21–2.99 (1H, m, H-3b), 2.84–2.70 (1H, m, H-4a), 2.68–2.58 (1H, m, H-4b), 2.00–1.82 (2H, m, H_2_-10), 1.81–1.73 (2H, m, H_2_-12), 1.56–1.44 (2H, m, H_2_-11), 1.45 (9H, br s, Boc); ^13^C NMR (CDCl_3_, 100 MHz) δ 168.8 (C-15), 155.3 (Boc), 137.0 (C-8a), 135.6 (C-9a), 135.1 (C-9a), 134.2 (C-17/C-18), 132.1 (C-16), 125.9 (C-4b), 123.4 (C-17/C-18), 122.7 (C-6), 119.4 (C-5), 115.0 (C-7), 114.0 (C-8), 109.3 (C-4a), 108.6 (C-4a), 80.2 (Boc), 51.4 (C-1), 50.6 (C-1), 39.0 (C-3), 37.8 (C-3), 37.3 (C-13), 33.6 (C-10), 28.6 (Boc), 28.0 (C-12), 23.3 (C-11), 21.5 (C-4); (+)-HRESIMS *m*/*z* 574.1312 [M + Na]^+^ (calcd for C_28_H_30_^79^BrN_3_NaO_4_, 574.1312), 576.1292 (calcd for C_28_H_30_^81^BrN_3_NaO_4_, 576.1291).

#### 3.2.9. *tert*-Butyl 1-(4-(1,3-Dioxoisoindolin-2-yl)butyl)-1,3,4,9-tetrahydro-2*H*-pyrido[3,4-*b*]indole-2-carboxylate (**21**)

To a solution of **17** (99 mg, 0.26 mmol) in MeOH (5 mL) was added (Boc)_2_O (64 mg, 0.29 mmol) and Et_3_N (0.110 mL, 0.79 mmol). The reaction mixture was stirred for 18 h at r.t. under N_2_ and then the solvent was removed *in vacuo*. Product purification was achieved using silica gel column chromatography (100% CH_2_Cl_2_ –>1% MeOH:CH_2_Cl_2_) to afford **21** as a beige gum (93 mg, 76%). R*_f_* = 0.90 (MeOH:CH_2_Cl_2_, 1:9); IR (ATR) *v*_max_ 3330, 2940, 1709, 1397, 1166 cm^−1^; ^1^H NMR (CDCl_3_, 400 MHz) δ 7.87–7.80 (2H, m, H-17/H-18), 7.74–7.68 (2H, m, H-17/H-18), 7.45 (1H, d, *J* = 7.7 Hz, H-5), 7.34 (1H, d, *J* = 8.0 Hz, H-8), 7.14 (1H, t, *J* = 7.8 Hz, H-7), 7.08 (1H, t, *J* = 7.8 Hz, H-6), 5.35–5.08 (1H, m, H-1), 4.56–4.22 (1H, m, H-3a), 3.80–3.65 (2H, m, H_2_-13), 3.23–3.02 (1H, m, H-3b), 2.88–2.74 (1H, m, H-4a), 2.72–2.62 (1H, m, H-4b), 2.01–1.82 (2H, m, H_2_-10), 1.80–1.73 (2H, m, H_2_-12), 1.59–1.49 (2H, m, H_2_-11), 1.44 (9H, br s, Boc); ^13^C NMR (CDCl_3_, 100 MHz) δ 168.7 (C-15), 155.3 (Boc), 136.1 (C-8a), 134.9 (C-9a), 134.1 (C-17/C-18), 132.2 (C-16), 127.0 (C-4b), 123.4 (C-17/C-18), 121.8 (C-7), 119.6 (C-6), 118.1 (C-5), 111.0 (C-8), 108.6 (C-4a), 80.0 (Boc), 51.6 (C-1), 50.8 (C-1), 39.0 (C-3), 37.9 (C-3), 37.4 (C-13), 34.2 (C-10), 33.8 (C-10), 28.6 (Boc), 28.2 (C-12), 23.4 (C-11), 21.7 (C-4), 21.4 (C-4); (+)-HRESIMS *m*/*z* 496.2208 [M + Na]^+^ (calcd for C_28_H_31_N_3_NaO_4_, 496.2207).

#### 3.2.10. *tert*-Butyl (*E*)-1-(4-(2,3-Bis(*tert*-butoxycarbonyl)guanidino)butyl)-7-bromo-1,3,4,9-tetrahydro-2*H*-pyrido[3,4-*b*]indole-2-carboxylate (**22**)

Following general procedure A, phthalimide-protected compound **20** (104 mg, 0.19 mmol) was stirred in a solution of EtOH (5 mL) and hydrazine hydrate (0.140 mL, 2.8 mmol). After workup, the crude reaction product was reacted, following general procedure B, with *N,N*′-di-Boc-1*H*-pyrazole-1-carboxamidine (**15**) (65 mg, 0.21 mmol) and DIPEA (0.160 mL, 1.0 mmol) in DMF (1 mL) to afford, after workup and chromatography, **22** as a pale-yellow oil/gum (37 mg, 29%). R*_f_* = 0.87 (MeOH:CH_2_Cl_2_, 1:9); IR (ATR) *v*_max_ 3326, 2976, 2931, 1719, 1639, 1615, 1365, 1156 cm^−1^; ^1^H NMR (CDCl_3_, 400 MHz) δ 11.49 (1H, br s, NH-14/NH-16), 8.48–8.34 (2H, m, NH-9, NH-14/NH-16), 7.46 (1H, d, *J* = 1.5 Hz, H-8), 7.30 (1H, d, *J* = 8.2 Hz, H-5), 7.17 (1H, dd, *J* = 8.2, 1.5 Hz, H-6), 5.34–5.09 (1H, m, H-1), 4.55–4.22 (1H, m, H-3a), 3.55–3.33 (2H, m, H_2_-13), 3.21–3.02 (1H, m, H-3b), 2.85–2.72 (1H, m, H-4a), 2.68–2.60 (1H, m, H-4b), 1.93–1.76 (2H, m, H_2_-10), 1.73–1.60 (2H, m, H_2_-12), 1.60–1.48 (2H, m, H_2_-11), 1.49 (9H, br s, Boc), 1.47 (18H, br s, Boc); ^13^C NMR (CDCl_3_, 100 MHz) δ 163.6 (C-15/Boc), 156.4 (C-15/Boc), 155.3 (C-15/Boc), 154.9 (C-15/Boc), 153.5 (C-15/Boc), 137.0 (C-8a), 135.7 (C-9a), 135.4 (C-9a), 125.9 (C-4b), 122.6 (C-6), 119.3 (C-5), 115.0 (C-7), 114.1 (C-8), 109.0 (C-4a), 108.5 (C-4a), 83.3 (Boc), 80.1 (Boc), 79.5 (Boc), 51.4 (C-1), 50.5 (C-1), 40.7 (C-13), 38.8 (C-3), 34.4 (C-10), 34.0 (C-10), 28.7 (C-12), 28.6 (Boc), 28.4 (Boc), 28.2 (Boc), 23.5 (C-11), 21.5 (C-4), 21.2 (C-4); (+)-HRESIMS *m*/*z* 664.2699 [M + H]^+^ (calcd for C_31_H_47_^79^BrN_5_O_6_, 664.2704), 666.2677 (calcd for C_31_H_47_^81^BrN_5_O_6_, 666.2684).

#### 3.2.11. *tert*-Butyl (*E*)-1-(4-(2,3-Bis(*tert*-butoxycarbonyl)guanidino)butyl)-1,3,4,9-tetrahydro-2*H*-pyrido[3,4-*b*]indole-2-carboxylate (**23**)

Following general procedure A, phthalimide-protected compound **21** (78 mg, 0.165 mmol) was stirred in a solution of EtOH (5 mL) and hydrazine hydrate (0.120 mL, 2.4 mmol). After workup, the crude reaction product was reacted, following general procedure B, with *N,N*′-di-Boc-1*H*-pyrazole-1-carboxamidine (56 mg, 0.18 mmol) and DIPEA (0.140 mL, 0.90 mmol) in DMF (1 mL) to afford, after workup and chromatography, **23** as a pale-yellow oil/gum (64 mg, 66% over two steps). R*_f_* = 0.93 (MeOH:CH_2_Cl_2_, 1:9); IR (ATR) *v*_max_ 3326, 2976, 2931, 1638, 1613, 1412, 1129 cm^−1^; ^1^H NMR (CDCl_3_, 400 MHz) δ 11.50 (1H, br s, NH-14/16), 8.36–8.13 (2H, m, NH-9, NH-14/NH-16), 7.46 (1H, d, *J* = 8.0 Hz, H-5), 7.32 (1H, d, *J* = 8.0 Hz, H-8), 7.14 (1H, t, *J* = 8.0 Hz, H-7), 7.08 (1H, t, *J* = 8.0 Hz, H-6), 5.37–5.09 (1H, m, H-1), 4.55–4.22 (1H, m, H-3a), 3.54–3.33 (2H, m, H_2_-13), 3.22–3.01 (1H, m, H-3b), 2.87–2.75 (1H, m, H-4a), 2.72–2.62 (1H, m, H-4b), 1.88–1.78 (2H, m, H_2_-10), 1.68–1.59 (2H, m, H_2_-12), 1.56–1.48 (2H, m, H_2_-11), 1.49 (9H, br s, Boc), 1.48 (18H, br s, Boc); ^13^C NMR (CDCl_3_, 100 MHz) δ 163.7 (C-15/Boc), 156.3 (C-15/Boc), 155.4 (C-15/Boc), 155.0 (C-15/Boc), 153.5 (C-15/Boc), 136.1 (C-8a), 134.9 (C-9a), 134.5 (C-9a), 127.0 (C-4b), 121.8 (C-7), 119.5 (C-6), 118.1 (C-5), 111.0 (C-8), 108.9 (C-4a), 108.4 (C-4a), 83.3 (Boc), 80.1 (Boc), 80.0 (Boc), 79.5 (Boc), 51.5 (C-1), 50.7 (C-1), 38.9 (C-3), 37.6 (C-3), 34.6 (C-10), 34.2 (C-10), 28.9 (C-12), 28.6 (Boc), 28.4 (Boc), 28.2 (Boc), 23.6 (C-11), 21.7 (C-4), 21.3 (C-4); (+)-HRESIMS *m*/*z* 586.3597 [M + H]^+^ (calcd for C_31_H_48_N_5_O_6_, 586.3599).

#### 3.2.12. (±)1-(4-(7-Bromo-2,3,4,9-tetrahydro-1*H*-pyrido[3,4-*b*]indol-1-yl)butyl)guanidine Bis(2,2,2 Trifluoroacetate) (**3**) ((±)-7-Bromohomotrypargine)

Following general procedure C, **22** (20 mg, 0.030 mmol) was reacted with TFA/CH_2_Cl_2_ for 4 h followed by workup and chromatography to afford the di-TFA salt **3** as a pale-yellow oil (13.8 mg, 78%). ^1^H NMR (CD_3_OD, 400 MHz) δ 7.53 (1H, d, *J* = 1.5 Hz, H-8), 7.41 (1H, d, *J* = 8.5 Hz, H-5), 7.18 (1H, dd, *J* = 8.5, 1.5 Hz, H-6), 4.71 (1H, dd, *J* = 8.5, 4.2 Hz, H-1), 3.73 (1H, dt, *J* = 12.6, 4.8 Hz, H-3a), 3.50–3.43 (1H, m, H-3b), 3.24 (2H, t, *J* = 6.9 Hz, H_2_-13), 3.09–3.05 (2H, m, H_2_-4), 2.32–2.23 (1H, m, H-10a), 2.08–1.96 (1H, m, H-10b), 1.79–1.69 (2H, m, H_2_-12), 1.69–1.61 (2H, m, H_2_-11); ^13^C NMR (CD_3_OD, 100 MHz) δ 158.7 (C-15), 139.1 (C-8a), 131.1 (C-9a), 126.4 (C-4b), 123.9 (C-6), 120.6 (C-5), 116.9 (C-7), 115.3 (C-8), 107.8 (C-4a), 54.5 (C-1), 42.8 (C-3), 42.1 (C-13), 32.8 (C-10), 29.7 (C-12), 23.3 (C-11), 19.4 (C-4); (+)-HRESIMS *m*/*z* 364.1132 [M+H]^+^ (calcd for C_16_H_23_^79^BrN_5_, 364.1131), 366.1108 (calcd for C_16_H_23_^81^BrN_5_, 366.1111). NMR data agreed with those reported in the literature [17].

#### 3.2.13. (±)-1-(4-(2,3,4,9-Tetrahydro-1*H*-pyrido[3,4-*b*]indol-1-yl)butyl)guanidine Bis(2,2,2 Trifluoroacetate) (**24**) ((±)-Homotrypargine)

Following general procedure C, Boc-protected compound **23** (25 mg, 0.043 mmol) was reacted with TFA/CH_2_Cl_2_ for 4 h followed by workup and chromatography to afford the di-TFA salt **24** as a yellow oil (14.9 mg, 67%). R*_f_* = 0.84 (RP-_18_, 10% HCl:MeOH, 5:95); IR (ATR) *v*_max_ 3353, 3191, 2978, 1671, 1201, 1135 cm^−1^; ^1^H NMR (CD_3_OD, 400 MHz) δ 7.48 (1H, d, *J* = 7.8 Hz, H-5), 7.36 (1H, d, *J* = 8.2 Hz, H-8), 7.16 (1H, ddd, *J* = 8.2, 7.8, 1.5 Hz, H-7), 7.06 (1H, ddd, *J* = 8.2, 7.8, 1.5 Hz, H-6), 4.73 (1H, dd, *J* = 8.8, 4.5 Hz, H-1), 3.74 (1H, dt, *J* = 12.6, 5.0 Hz, H-3a), 3.52–3.44 (1H, m, H-3b), 3.25 (2H, t, *J* = 6.8 Hz, H_2_-13), 3.15–3.02 (2H, m, H_2_-4), 2.34–2.24 (1H, m, H-10a), 2.07–1.97 (1H, m, H-10b), 1.78–1.69 (2H, m, H_2_-12), 1.69–1.60 (2H, m, H_2_-11); ^13^C NMR (CD_3_OD, 100 MHz) δ 158.7 (C-15), 138.3 (C-8a), 129.9 (C-9a), 127.4 (C-4b), 123.6 (C-7), 120.7 (C-6), 119.1 (C-5), 112.3 (C-8), 107.4 (C-4a), 54.7 (C-1), 42.9 (C-3), 42.1 (C-13), 33.0 (C-10), 29.7 (C-12), 23.3 (C-11), 19.5 (C-4); (+)-HRESIMS *m/z* 286.2025 [M + H]^+^ (calcd for C_16_H_24_N_5_, 286.2026).

### 3.3. Biological Assays

See Supplementary File for biological assay protocols (Protocol S1–S4) [20].

## Figures and Tables

**Table 1:** Intrinsic antimicrobial activity (μM) of opacaline natural products and related des bromo analogues.

Compound	R	Capping on Side Chain	Intrinsic Antimicrobial Activity (μM)		
			*P. aeruginosa* ^a^	*E. coli* ^b^	*S. aureus* ^c^
Opacaline A (**1**) ^d^	Br	Guanidine	211	105	6.6
Opacaline B (**2**) ^d^	Br	Guanidine	100	100	25
**3** ^d^	Br	Guanidine	800	400	50
**4** ^d^	Br	Guanidine	328	328	10
**5**	H	Guanidine	506	>506	506
**6**	H	Phthalimide	>541	>541	>541
**7**	H	Amine	>566	>566	566
**8**	H	Boc guanidine	>415	>415	>415

^a^ *P. aeruginosa* PAO1 with MIC values presented as the mean (*n* = 3); ^b^ *E. coli* ATCC 25922 with MIC values presented as the mean (*n* = 3); ^c^ *S. aureus* ATCC 25923 with MIC values presented as the mean (*n* = 3); ^d^ natural product isolated from *Pseudodistoma opacum*.

**Table 2:** Antibiotic-potentiating activity (μM) of opacaline natural products and related des bromo analogues.

Compound	R	Capping on Side Chain	Antibiotic Potentiating Activity (μM)			
			*P.a.* ^a^	*P.a.* Dox. ^b^ (Fold)	*E.c.* ^c^	*E.c.* Eryth. ^d^ (Fold)
Opacaline A (**1**) ^e^	Br	Guanidine	211	6.59 (32)	105	6.59 (16)
Opacaline B (**2**) ^e^	Br	Guanidine	100	12.5 (8)	100	25 (4)
**3** ^e^	Br	Guanidine	800	50 (16)	400	200 (2)
**4** ^e^	Br	Guanidine	328	41 (8)	328	-
**5**	H	Guanidine	506	126 (4)	>506	>506 (0)
**6**	H	Phthalimide	>541	>541 (0)	>541	>541 (0)
**7**	H	Amine	>566	283 (2)	>566	283 (2)
**8**	H	Boc guanidine	>415	>415 (0)	>415	>415 (0)

^a^ *P. aeruginosa* PAO1 with MIC values presented as the mean (*n* = 3); ^b^ potentiating activity in combination with doxycycline at 2 μg/mL against *P. aeruginosa* PAO1. Fold change shown in parentheses is the ratio between the intrinsic MIC of the test compound and the combination MIC; ^c^ *E. coli* ATCC 25922 with MIC values presented as the mean (*n* = 3); ^d^ potentiating activity in combination with erythromycin at 2 μg/mL against *E. coli* ATCC 25922. Fold change shown in parentheses is the ratio between the intrinsic MIC of the test compound and the combination MIC; ^e^ natural product isolated from *Pseudodistoma opacum*.

**Table 3:** Intrinsic antimicrobial activity (μM) of synthetic compounds.

Compound	R	Capping on Side Chain	Intrinsic Antimicrobial Activity (μM)		
			*P. aeruginosa* ^a^	*E. coli* ^b^	*S. aureus* ^c^
**11**	Br	Phthalimide	>353	>353	44
**13**	Br	Phthalimide	>446	446	>446
**14**	Br	Amine	462	>462	116
**16**	Br	Boc guanidine	>357	>357	>357
**17**	H	Phthalimide	>410	>410	>410
**18**	Br	Amine	363	363	182
**19**	H	Amine	>424	>424	>424
**20**	Br	Phthalimide	>362	>362	>362
**21**	H	Phthalimide	>422	>422	>422
**22**	Br	Boc guanidine	>301	>301	>301
**23**	H	Boc guanidine	>341	>341	>341
**3** (synthetic)	Br	Guanidine	>338	>338	84
**24**	H	Guanidine	>390	>390	>390

^a^ *P. aeruginosa* PAO1 with MIC values presented as the mean (*n* = 3); ^b^ *E. coli* ATCC 25922 with MIC values presented as the mean (*n* = 3); ^c^ *S. aureus* ATCC 25923 with MIC values presented as the mean (*n* = 3).

**Table 4:** Antibiotic-potentiating activity (μM) of synthetic compounds.

Compound	R	Capping on Side Chain	Antibiotic-Potentiating Activity (μM)			
			*P.a.* ^a^	*P.a.* Dox. ^b^ (Fold)	*E.c.* ^c^	*E.c.* Eryth. ^d^ (Fold)
**11**	Br	Phthalimide	>353	>353 (0)	>353	>353 (0)
**13**	Br	Phthalimide	>446	>446 (0)	446	>446 (0)
**14**	Br	Amine	462	116 (4)	>462	116 (4)
**16**	Br	Boc guanidine	>357	>357 (0)	>357	>357 (0)
**17**	H	Phthalimide	>410	205 (2)	>410	205 (2)
**18**	Br	Amine	363	45 (8)	363	91 (4)
**19**	H	Amine	>424	>424 (0)	>424	106 (4)
**20**	Br	Phthalimide	>362	>362 (0)	>362	>362 (0)
**21**	H	Phthalimide	>422	>422 (0)	>422	13 (32)
**22**	Br	Boc guanidine	>301	>301 (0)	>301	>301 (0)
**23**	H	Boc guanidine	>341	>341 (0)	>341	>341 (0)
**3** (synthetic)	Br	Guanidine	>338	42 (8)	>338	21 (16)
**24**	H	Guanidine	>390	>390 (0)	>390	>390 (0)

^a^ *P. aeruginosa* PAO1 with MIC values presented as the mean (*n* = 3); ^b^ potentiating activity in combination with doxycycline at 2 μg/mL against *P. aeruginosa* PAO1. Fold change shown in parentheses is the ratio between the intrinsic MIC of the test compound and the combination MIC; ^c^ *E. coli* ATCC 25922 with MIC values presented as the mean (*n* = 3); ^d^ potentiating activity in combination with erythromycin at 2 μg/mL against *E. coli* ATCC 25922. Fold change shown in parentheses is the ratio between the intrinsic MIC of the test compound and the combination MIC.

## Data Availability

Data are contained within the article or Supplementary Materials.

## Supplementary Materials

The following supporting information can be downloaded at: https://www.mdpi.com/article/10.3390/md24070248/s1, Protocol S1: Antimicrobial assays; Protocol S2: Determination of the MICs of antibiotics in the presence of synergising compounds; Protocol S3: Cytotoxicity assay; Protocol S4: Hemolysis assay; Figure S1: ^1^H NMR spectrum of natural product opacaline A **1** (CD_3_OD, 400 MHz); Figure S2: ^1^H NMR spectrum of natural product opacaline B **2** (CD_3_OD, 400 MHz); Figure S3: ^1^H NMR spectrum of natural product (−)-7-bromohomotrypargine (**3**) (CD_3_OD, 400 MHz); Figure S4: ^1^H NMR spectrum of natural product 7-bromo-*N*-hydroxyhomotrypargine (**4**) (CD_3_OD, 400 MHz); Figure S5: ^1^H NMR spectrum of compound **5** (CD_3_OD, 400 MHz; Figure S6: ^1^H NMR spectrum of compound **6** (CDCl_3_, 400 MHz); Figure S7: ^13^C NMR spectrum of compound **6** (CDCl_3_, 100 MHz); Figure S8: ^1^H NMR spectrum of compound **7** (CD_3_OD, 400 MHz); Figure S9: ^13^C NMR spectrum of compound **7** (CD_3_OD, 100 MHz); Figure S10: ^1^H NMR spectrum of compound **8** (CDCl_3_, 400 MHz); Figure S11: ^1^H NMR spectrum of compound **11** (CDCl_3_, 400 MHz); Figure S12: ^13^C NMR spectrum of compound **11** (CDCl_3_, 100 MHz); Figure S13: ^1^H NMR spectrum of compound **13** (CDCl_3_, 400 MHz); Figure S14: ^13^C NMR spectrum of compound **13** (CDCl_3_, 100 MHz); Figure S15: ^1^H NMR spectrum of compound **14** (CD_3_OD, 400 MHz); Figure S16: ^13^C NMR spectrum of compound **14** (CD_3_OD, 100 MHz); Figure S17: ^1^H NMR spectrum of compound **16** (CDCl_3_, 400 MHz); Figure S18: ^13^C NMR spectrum of compound **16** (CDCl_3_, 100 MHz); Figure S19: ^1^H NMR spectrum of opacaline A **1** (synthetic) (CD_3_OD, 400 MHz); Figure S20: ^1^H NMR spectrum of compound **18** (CD_3_OD, 400 MHz); Figure S21: ^13^C NMR spectrum of compound **18** (CD_3_OD, 100 MHz); Figure S22: ^1^H NMR spectrum of compound **19** (CD_3_OD, 400 MHz); Figure S23: ^13^C NMR spectrum of compound **19** (CD_3_OD, 100 MHz); Figure S24: ^1^H NMR spectrum of compound **20** (CDCl_3_, 400 MHz); Figure S25: ^1^H NMR spectrum of compound **21** (CDCl_3_, 400 MHz); Figure S26: ^1^H NMR spectrum of compound **22** (CDCl_3_, 400 MHz); Figure S27: ^1^H NMR spectrum of compound **23** (CDCl_3_, 400 MHz); Figure S28: ^1^H NMR spectrum of (±)7-bromohomotrypargine **3** (synthetic) (CD_3_OD, 400 MHz); Figure S29: ^1^H NMR spectrum of compound **24** (CD_3_OD, 400 MHz); Figure S30: ^13^C NMR spectrum of compound **24** (CD_3_OD, 100 MHz).
