# Supplementary material for: A Structure–Activity Relationship Study of Trypargine and Opacaline β-Carbolines
Source: Mar Drugs. 2026 Jul 17;24(7):248. doi: 10.3390/md24070248 (PMC13413296; doi:10.3390/md24070248)
Supplement: Supplementary file 1 [file marinedrugs-24-00248-s001.zip › marinedrugs-4431338-supplementary.pdf]

Supporting Information

A Structure-Activity Relationship Study of Trypargine and Opacaline β-Carbolines

Dan Chen [1], Florent Rouvier [2], Jean Michel Brunel [2], Brent R. Copp [1] and Melissa M. Cadelis [1,*]

[1] School of Chemical Sciences, The University of Auckland, Private Bag 92019, Auckland 1142, New Zealand
[2] Aix Marseille Univ, INSERM, SSA, MCT, Faculté de pharmacie, 27 bd Jean Moulin, 13385 Marseille, France
* Correspondence: m.cadelis@auckland.ac.nz

Contents

Protocol S1 Antimicrobial assays 3
Protocol S2 Determination of the MICs of antibiotics in the presence of synergising compounds 3
Protocol S3 Cytotoxicity assay 3
Protocol S4 Hemolysis assay 3
Figure S1 ^{1}H NMR spectrum of natural product opacaline A **1** (CD_3OD, 400 MHz) 4
Figure S2 ^{1}H NMR spectrum of natural product opacaline B **2** (CD_3OD, 400 MHz) 5
Figure S3 ^{1}H NMR spectrum of natural product (-)-7-bromohomotrypargine **3** (CD_3OD, 400 MHz) 6
Figure S4 ^{1}H NMR spectrum of natural product 7-bromo-*N*-hydroxyhomotrypargine **4** (CD_3OD, 400 MHz) 7
Figure S5 ^{1}H NMR spectrum of compound **5** (CD_3OD, 400 MHz) 8
Figure S6 ^{1}H NMR spectrum of compound **6** ($CDCl_3$, 400 MHz) 9
Figure S7 ^{13}C NMR spectrum of compound **6** ($CDCl_3$, 100 MHz) 10
Figure S8 ^{1}H NMR spectrum of compound **7** (CD_3OD, 400 MHz) 11
Figure S9 ^{13}C NMR spectrum of compound **7** (CD_3OD, 100 MHz) 12
Figure S10 ^{1}H NMR spectrum of compound **8** ($CDCl_3$, 400 MHz) 13
Figure S11 ^{1}H NMR spectrum of compound **11** ($CDCl_3$, 400 MHz) 14
Figure S12 ^{13}C NMR spectrum of compound **11** ($CDCl_3$, 100 MHz) 15
Figure S13 ^{1}H NMR spectrum of compound **13** ($CDCl_3$, 400 MHz) 16
Figure S14 ^{13}C NMR spectrum of compound **13** ($CDCl_3$, 100 MHz) 17
Figure S15 ^{1}H NMR spectrum of compound **14** (CD_3OD, 400 MHz) 18
Figure S16 ^{13}C NMR spectrum of compound **14** (CD_3OD, 100 MHz) 19

Figure S17 ^{1}H NMR spectrum of compound **16** ($CDCl_3$, 400 MHz) 20
Figure S18 ^{13}C NMR spectrum of compound **16** ($CDCl_3$, 100 MHz) 21
Figure S19 ^{1}H NMR spectrum of opacaline A **1** (synthetic) (CD_3OD, 400 MHz) 22
Figure S20 ^{1}H NMR spectrum of compound **18** (CD_3OD, 400 MHz) 23
Figure S21 ^{13}C NMR spectrum of compound **18** (CD_3OD, 100 MHz) 24
Figure S22 ^{1}H NMR spectrum of compound **19** (CD_3OD, 400 MHz) 25
Figure S23 ^{13}C NMR spectrum of compound **19** (CD_3OD, 100 MHz) 26
Figure S24 ^{1}H NMR spectrum of compound **20** ($CDCl_3$, 400 MHz) 27
Figure S25 ^{1}H NMR spectrum of compound **21** ($CDCl_3$, 400 MHz) 28
Figure S26 ^{1}H NMR spectrum of compound **22** ($CDCl_3$, 400 MHz) 29
Figure S27 ^{1}H NMR spectrum of compound **23** ($CDCl_3$, 400 MHz) 30
Figure S28 ^{1}H NMR spectrum of (±)7-bromohomotrypargine **3** (synthetic) (CD_3OD, 400 MHz) 31
Figure S29 ^{1}H NMR spectrum of compound **24** (CD_3OD, 400 MHz) 32
Figure S30 ^{13}C NMR spectrum of compound **24** (CD_3OD, 100 MHz) 33

Protocol S1 Antimicrobial assays

Antimicrobial evaluation against *Staphylococcus aureus* ATCC 25923, *E. coli* ATCC 25922 and *Pseudomonas aeruginosa* PAO1 was determined in microplates using the standard broth dilution method in accordance with the recommendations of the Comité de l'AntibioGramme de la Société Française de Microbiologie (CA-SFM). Briefly, the minimal inhibitory concentrations (MICs) were determined with an inoculum of 10^5 CFU in 200 µL of Mueller–Hinton broth (MHB) containing two-fold serial dilutions of each opacaline derivative. The MIC was defined as the lowest concentration of drug that completely inhibited visible growth after incubation for 18 h at 37 °C. To determine all MICs, the measurements were independently repeated in triplicate.

Protocol S2 Determination of the MICs of antibiotics in the presence of synergising compounds

Briefly, restoring enhancer concentrations were determined with an inoculum of 5×10^5 CFU in 200 µL of MHB containing two-fold serial dilutions of each opacaline derivative in the presence of doxycycline at 2 µg/mL for P; aeruginosa PA01 and erythromycine at 2 µg/mL for *E. coli* ATCC 25922. The lowest concentration of opacaline derivatives that completely inhibited visible growth after incubation for 18 h at 37 °C was determined. These measurements were independently repeated in triplicate.

Protocol S3 Cytotoxicity assay

HEK293 cells were counted manually in a Neubauer haemocytometer and plated at a density of 5,000 cells/well into each well of the 384-well plates containing the 25x (2 µL) concentrated compounds. The medium used was Dulbecco's modified eagle medium (DMEM) supplemented with 10% fetal bovine serum (FBS). Cells were incubated together with the compounds for 20 h at 37 ºC, 5% CO_2. To measure cytotoxicity, 5 µL (equals 100 µM final) of resazurin was added to each well after incubation, and incubated for further 3 h at 37 ºC with 5% CO_2. After final incubation fluorescence intensity was measured as Fex 560/10 nm, em 590/10 nm ($F_{560/590}$) using a Tecan M1000 Pro monochromator plate reader. CC_{50} values (concentration at 50% cytotoxicity) were calculated by normalizing the fluorescence readout, with 74 µg/mL tamoxifen as negative control (0%) and normal cell growth as positive control (100%). The concentration-dependent percentage cytotoxicity was fitted to a dose response function (using Pipeline Pilot) and CC_{50} values determined.

Protocol S4 Hemolysis assay

Human whole blood was washed three times with 3 volumes of 0.9% NaCl and then resuspended in same to a concentration of 0.5×10^8 cells/mL, as determined by manual cell count in a Neubauer haemocytometer. The washed cells were then added to the 384-well compound-containing plates for a final volume of 50 µL. After a 10 min shake on a plate shaker the plates were then incubated for 1 h at 37 ºC. After incubation, the plates were centrifuged at 1,000g for 10 min to pellet cells and debris, 25 µL of the supernatant was then transferred to a polystyrene 384-well assay plate. Haemolysis was determined by measuring the supernatant absorbance at 405 mm (OD_{405}). The absorbance was measured using a Tecan M1000 Pro monochromator plate reader. HC_{10} (concentration at 10% haemolysis) was calculated by curve fitting the inhibition values *vs.* log (concentration) using a sigmoidal dose-response function with variable fitting values for top, bottom and slope.

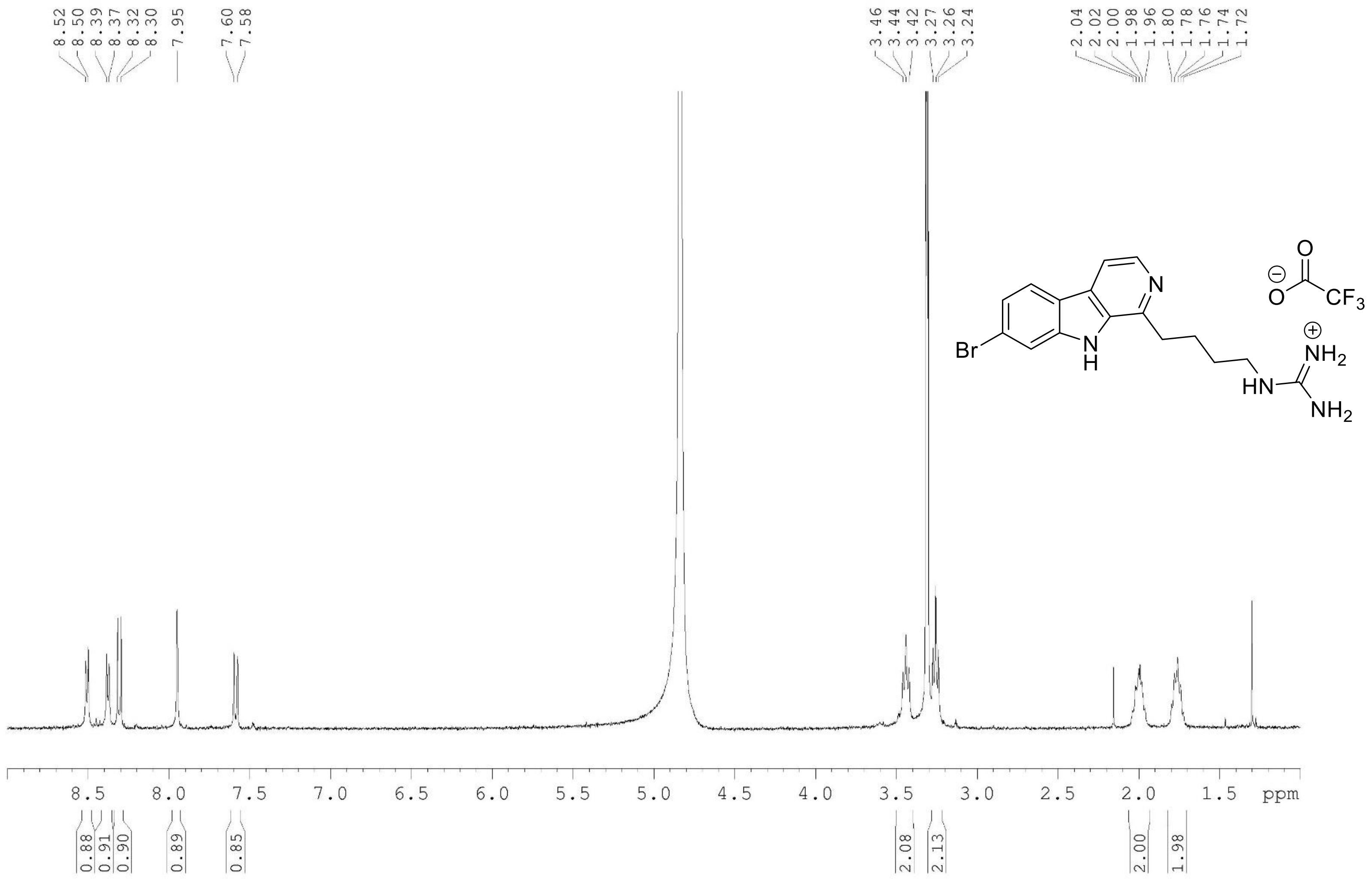

Figure S1 ^{1}H NMR spectrum of natural product opacaline A (**1**) (CD_3OD, 400 MHz).

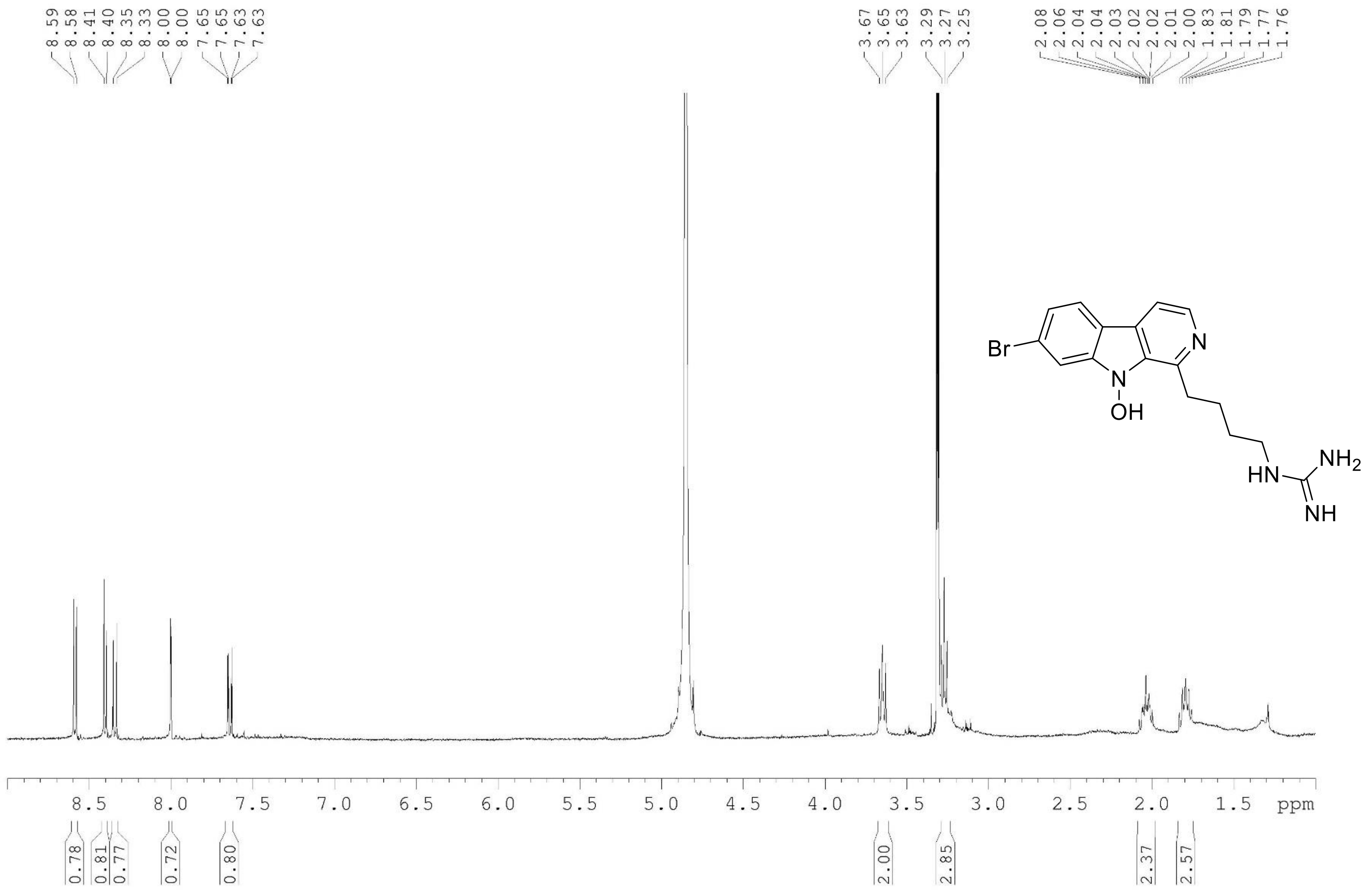

Figure S2 ^{1}H NMR spectrum of natural product opacaline B (**2**) (CD_3OD, 400 MHz).

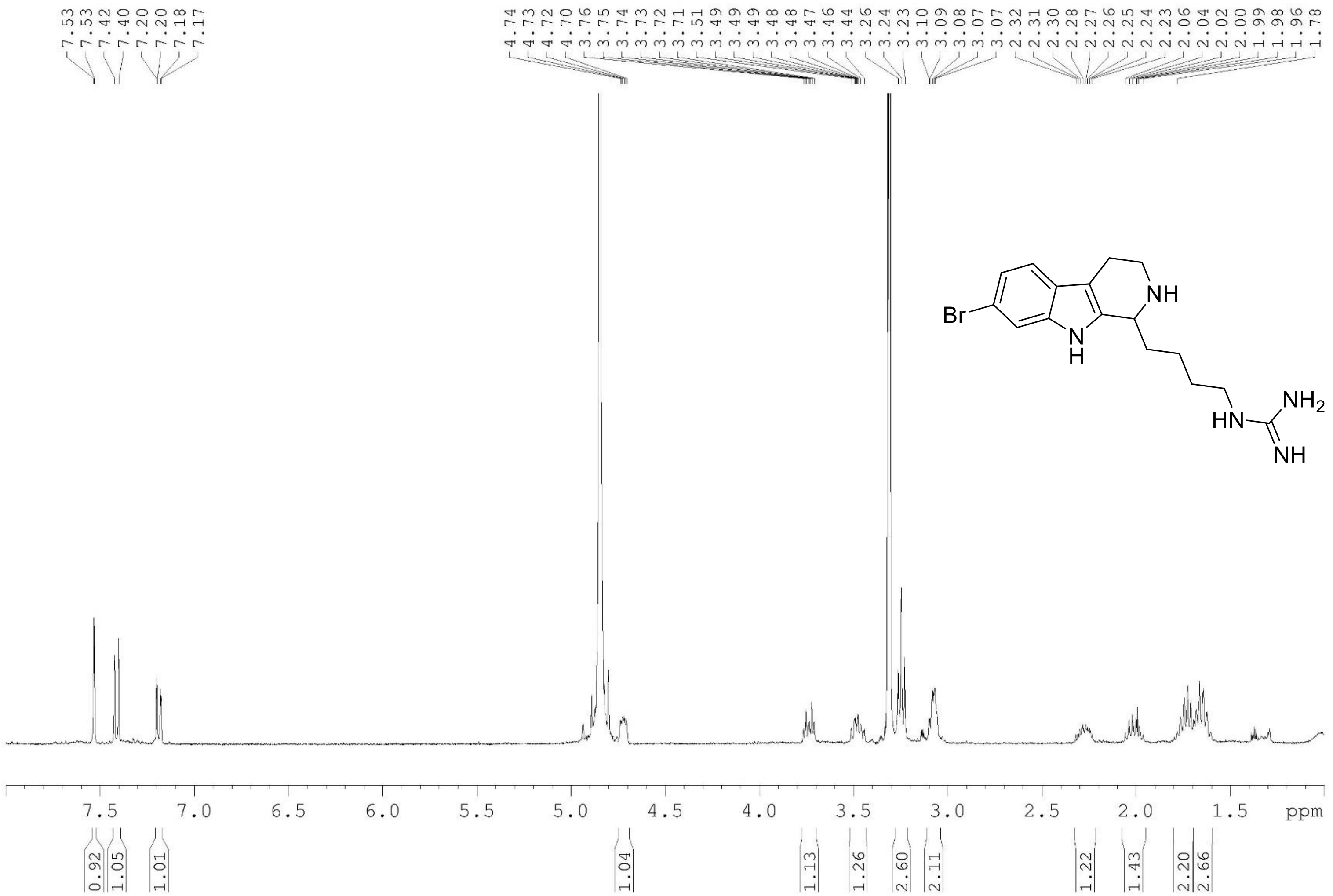

Figure S3 ^{1}H NMR spectrum of natural product (-)-7-bromohomotrypargine (**3**) (CD_3OD, 400 MHz).

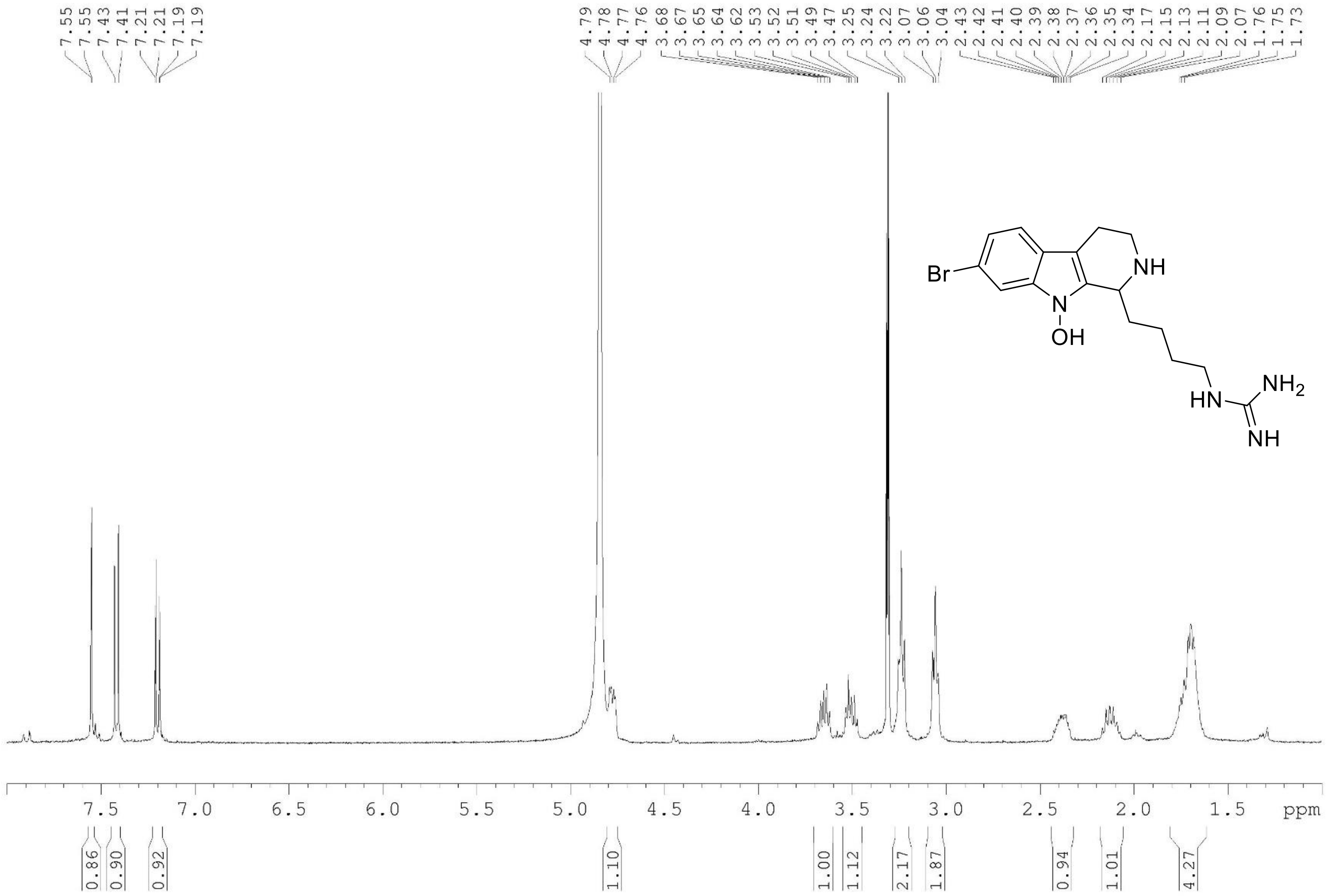

Figure S4 ^{1}H NMR spectrum of natural product 7-bromo-*N*-hydroxyhomotrypargine (**4**) (CD_3OD, 400 MHz).

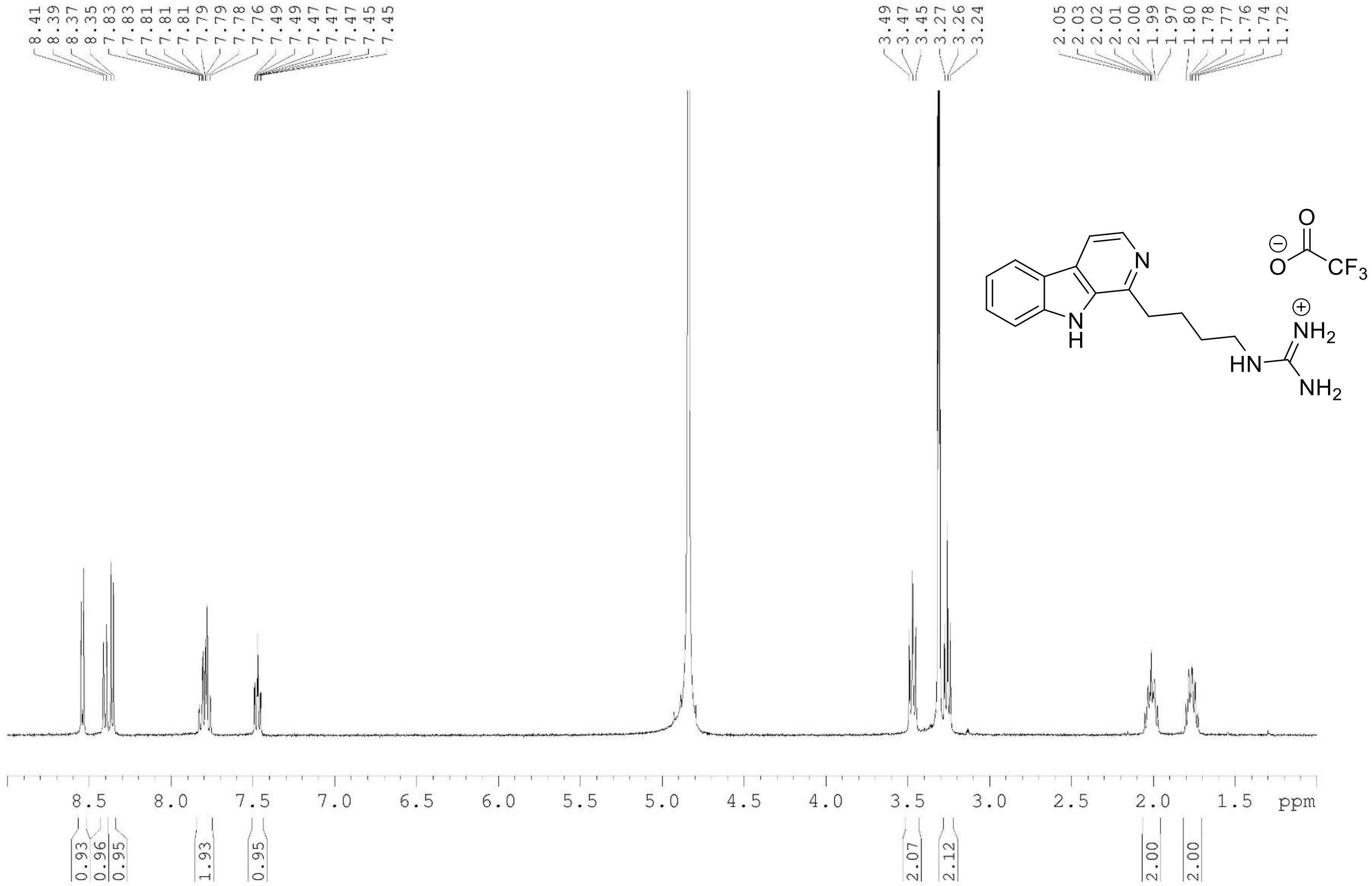

Figure S5 ^{1}H NMR spectrum of compound **5** (CD_3OD, 400 MHz).

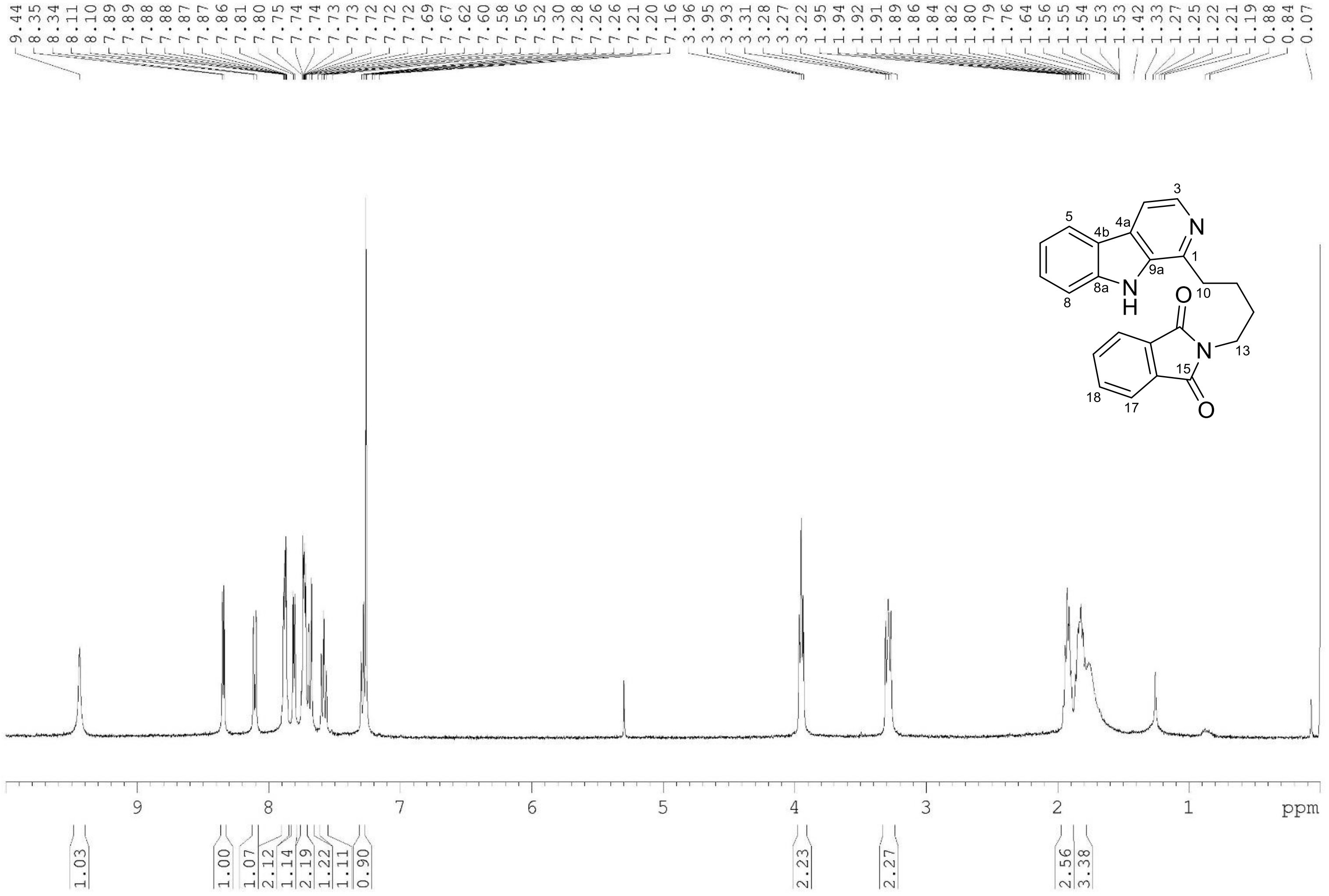

Figure S6 ^{1}H NMR spectrum of compound **6** ($CDCl_3$, 400 MHz).

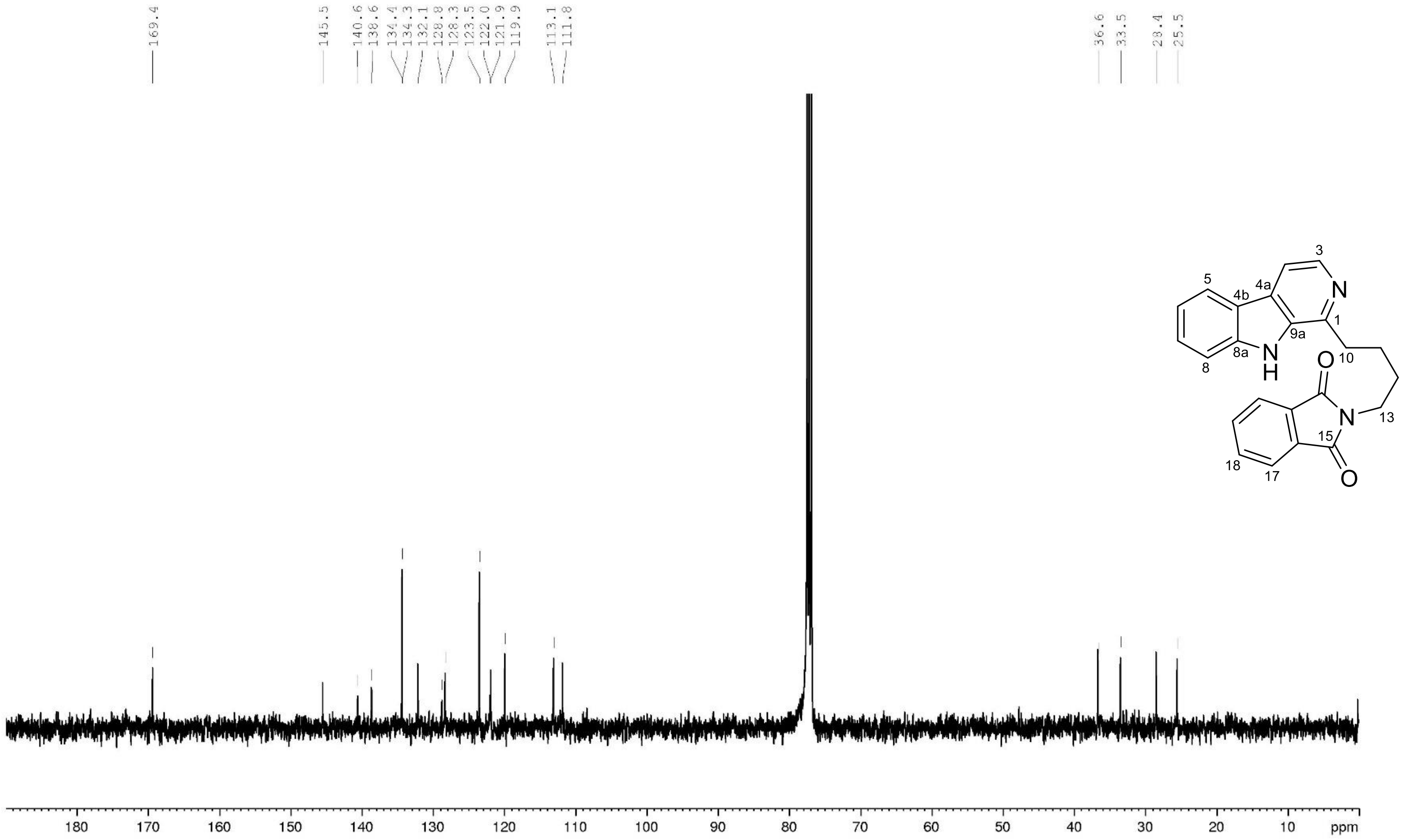

Figure S7 ^{13}C NMR spectrum of compound **6** ($CDCl_3$, 100 MHz)

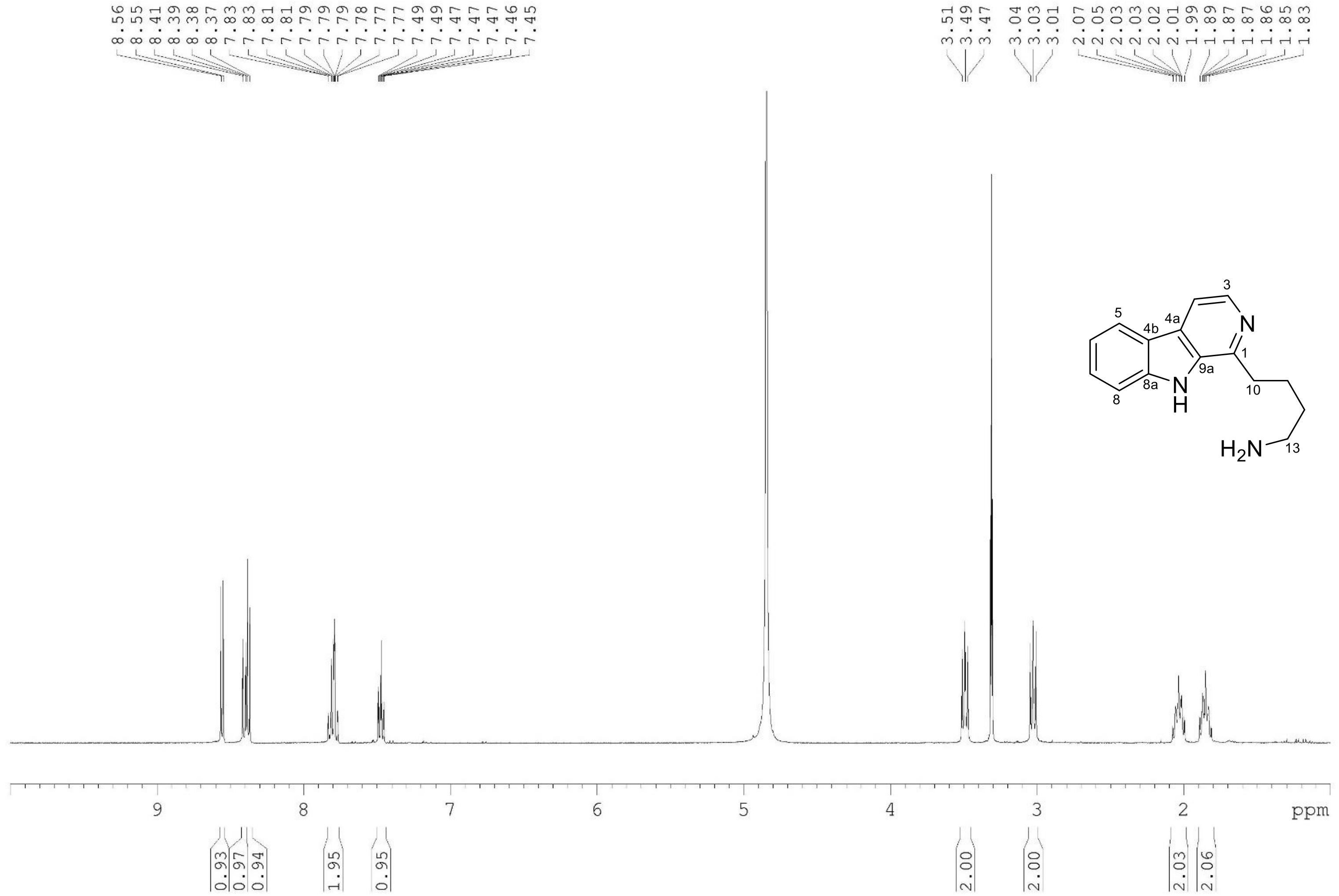

Figure S8 ^{1}H NMR spectrum of compound **7** (CD_3OD, 400 MHz).

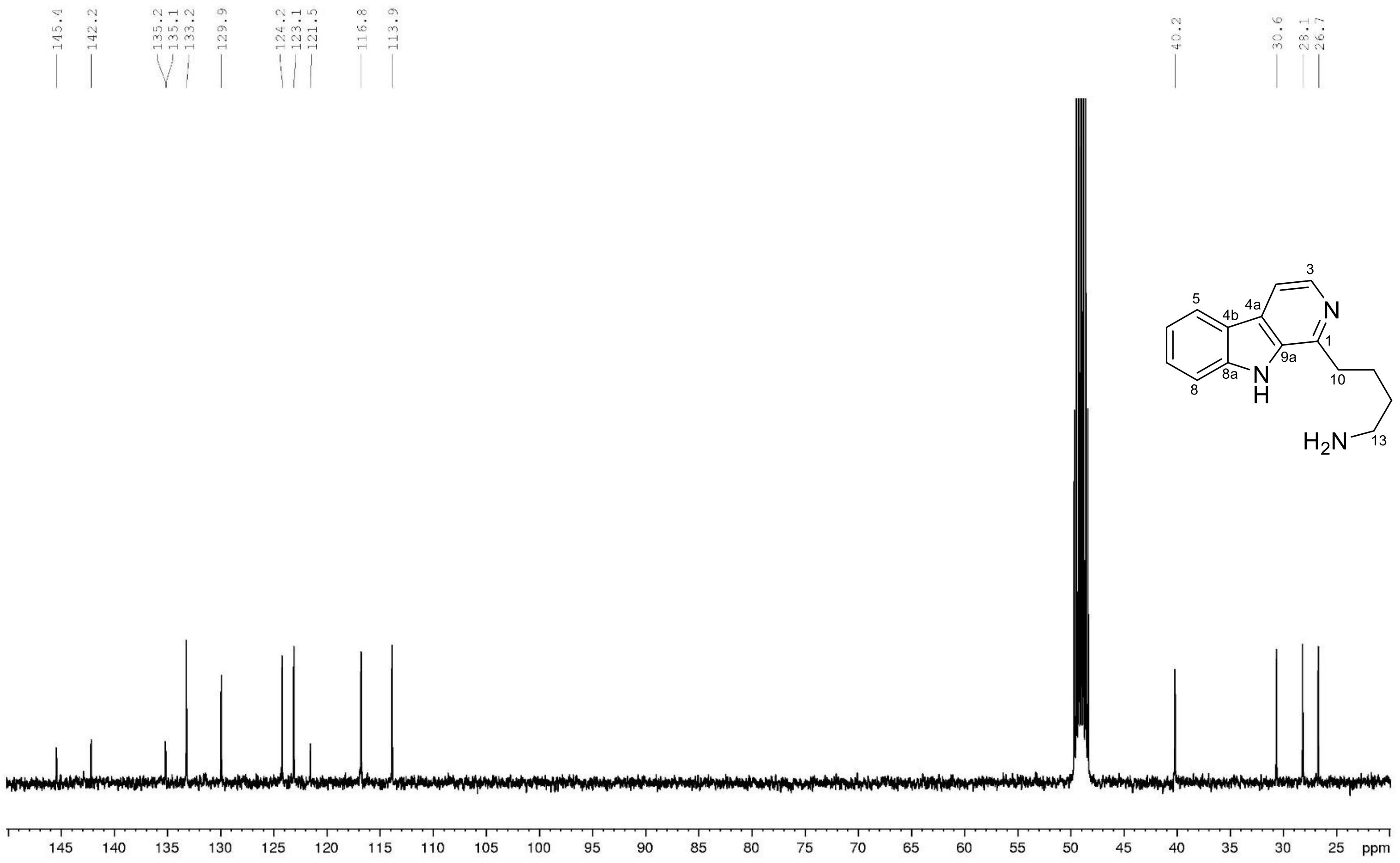

Figure S9 ^{13}C NMR spectrum of compound **7** (CD_3OD, 100 MHz)

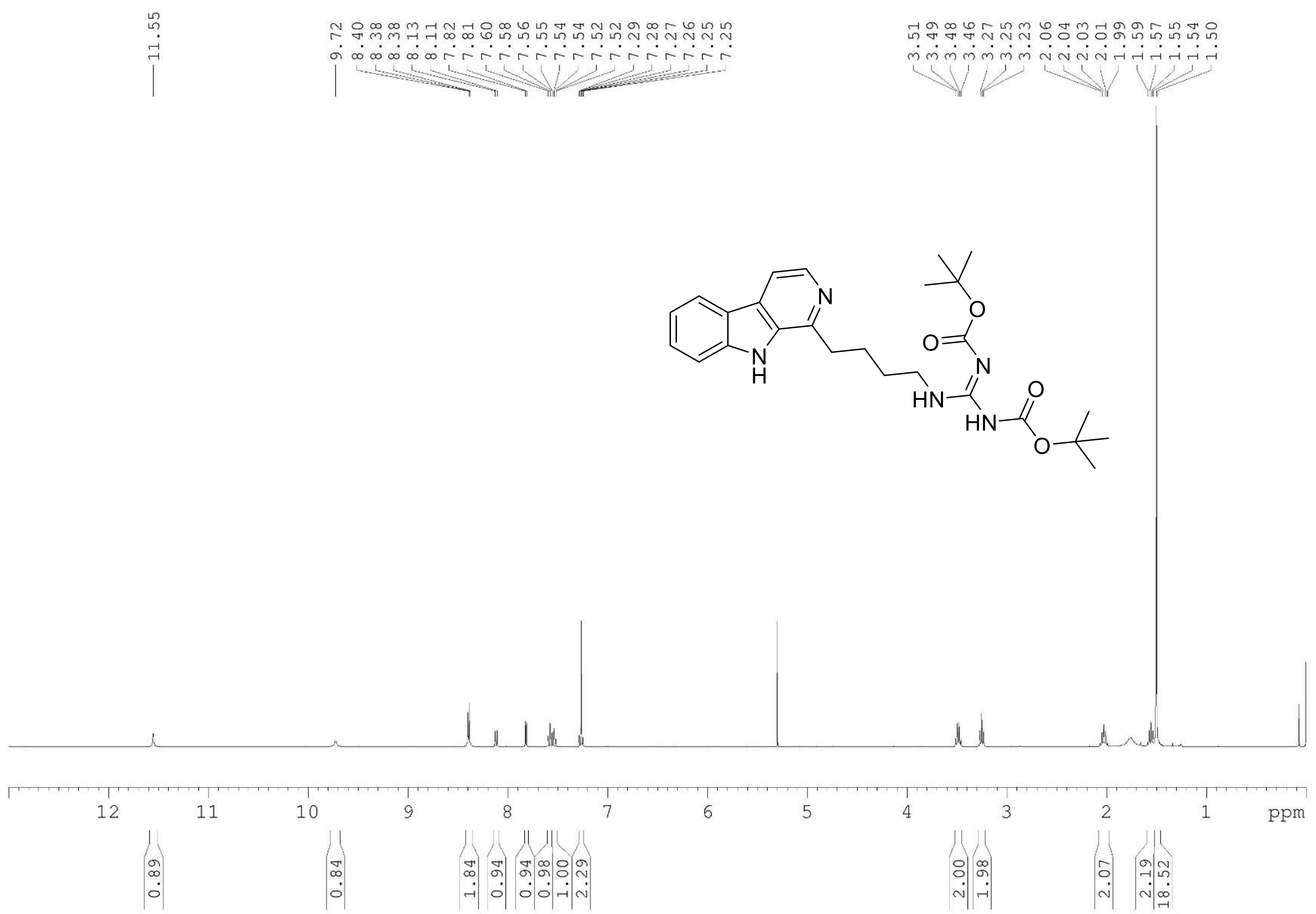

Figure S10 ^{1}H NMR spectrum of compound **8** ($CDCl_3$, 400 MHz).

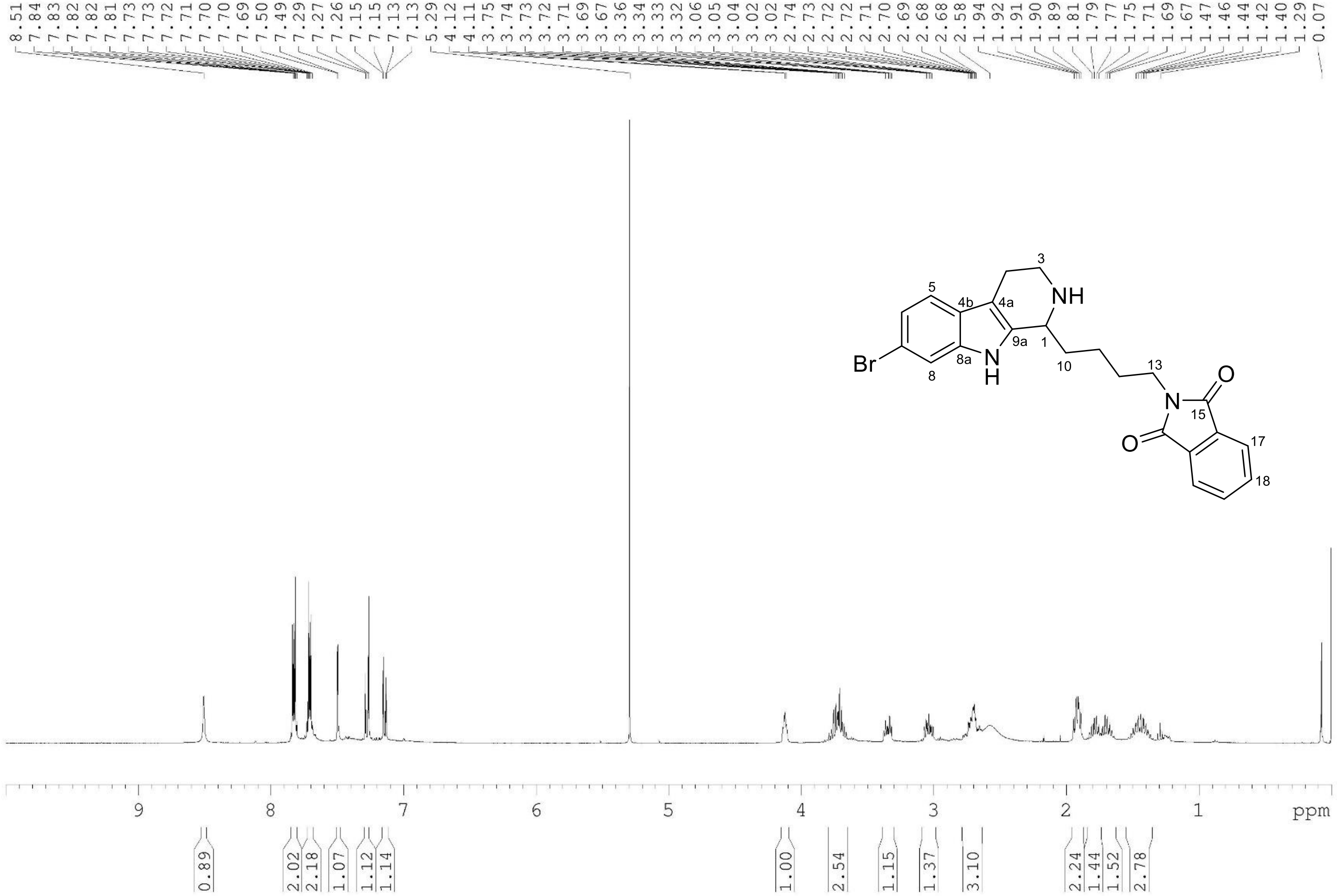

Figure S11 ^{1}H NMR spectrum of compound **11** ($CDCl_3$, 400 MHz).

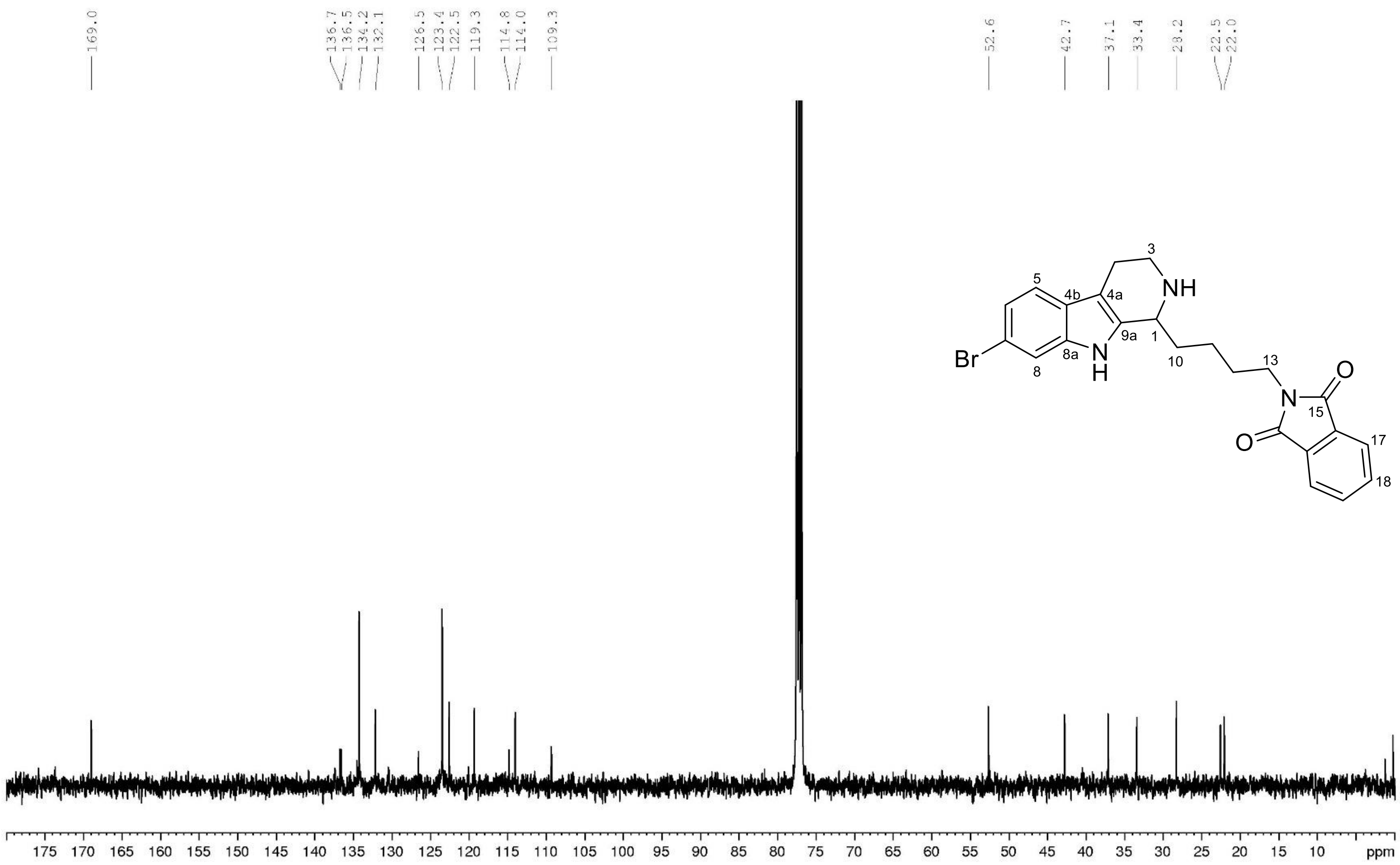

Figure S12 ^{13}C NMR spectrum of compound **11** ($CDCl_3$, 100 MHz).

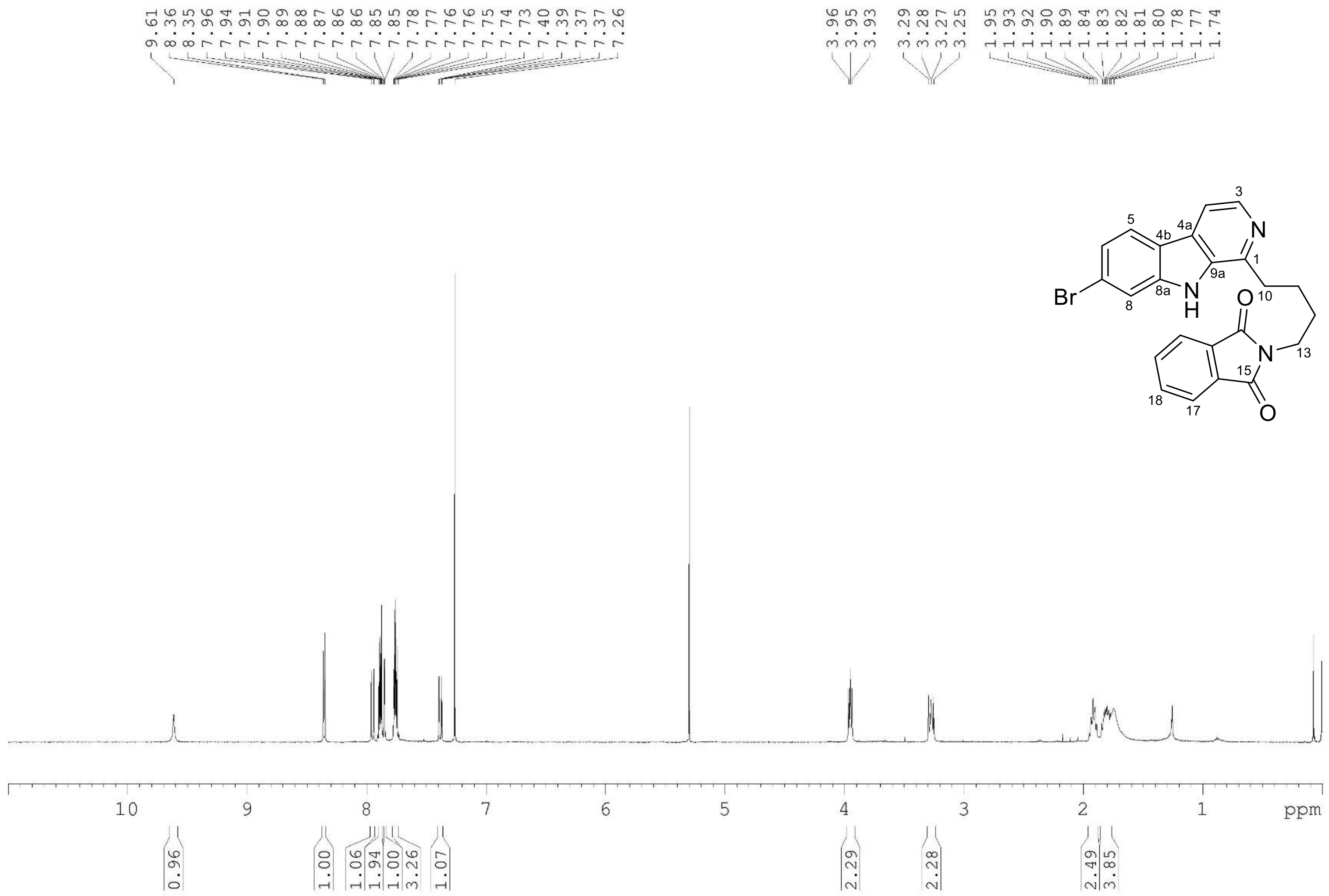

Figure S13 ^{1}H NMR spectrum of compound **13** ($CDCl_3$, 400 MHz).

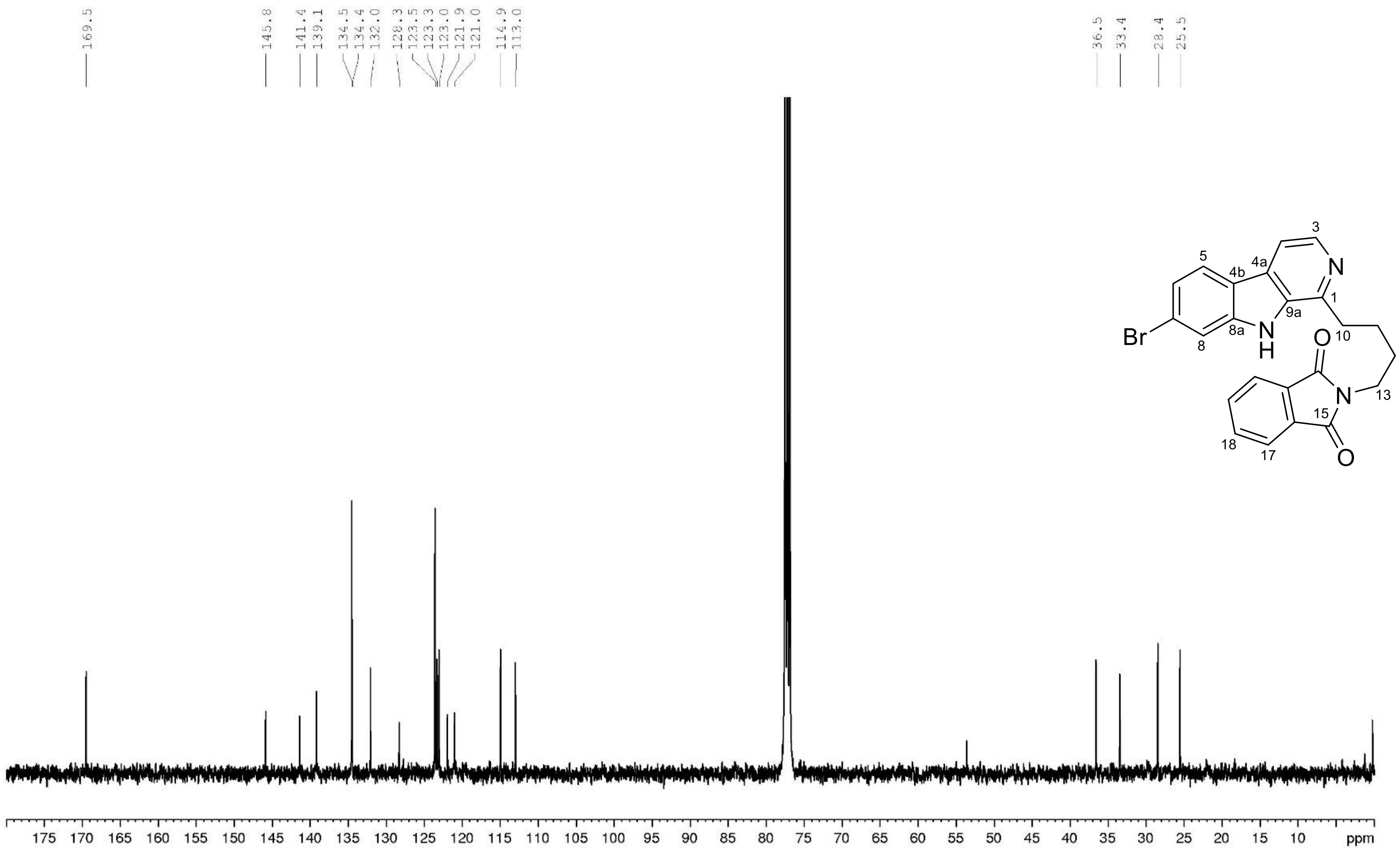

Figure S14 ^{13}C NMR spectrum of compound **13** ($CDCl_3$, 100 MHz).

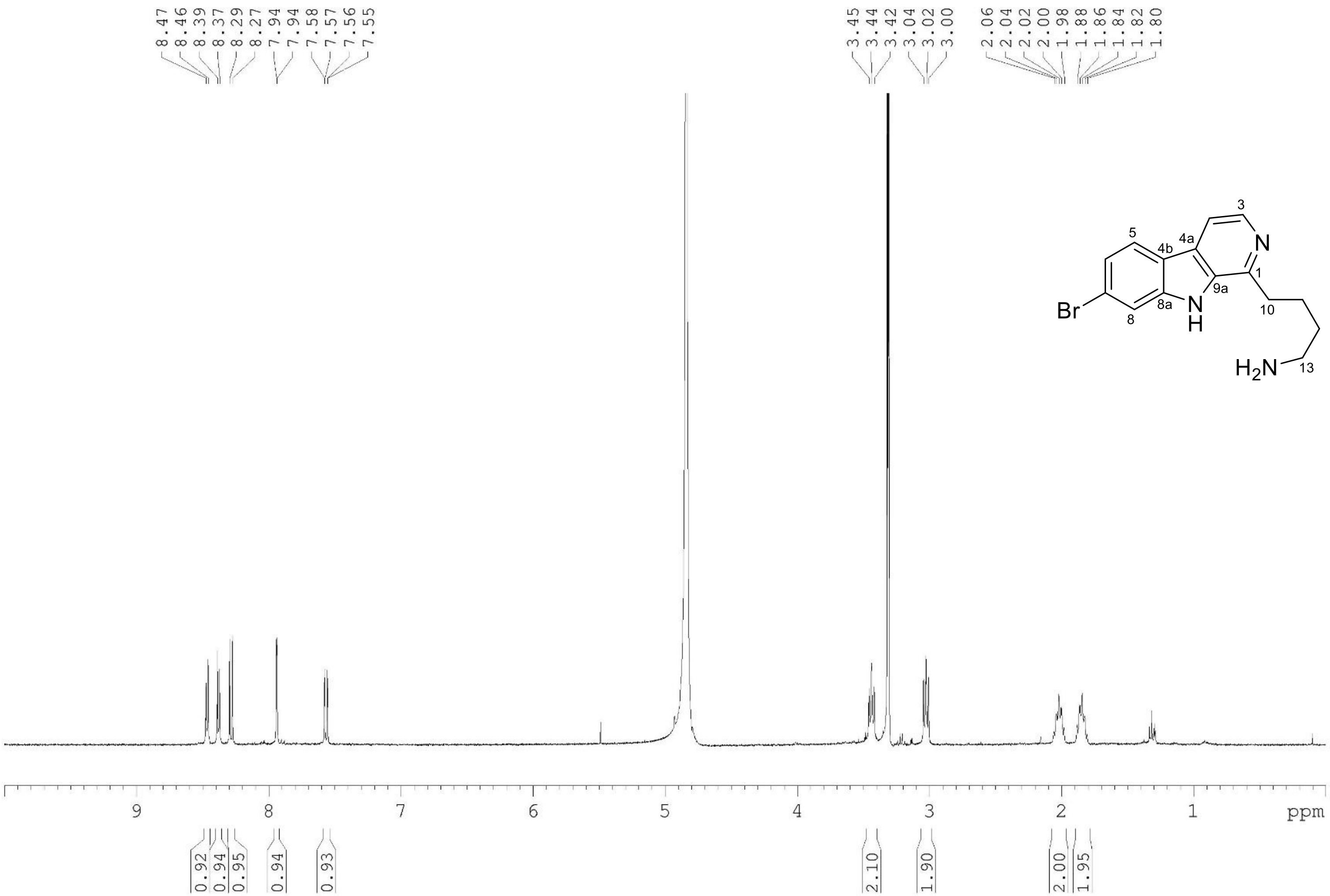

Figure S15 ^{1}H NMR spectrum of compound **14** (CD_3OD, 400 MHz).

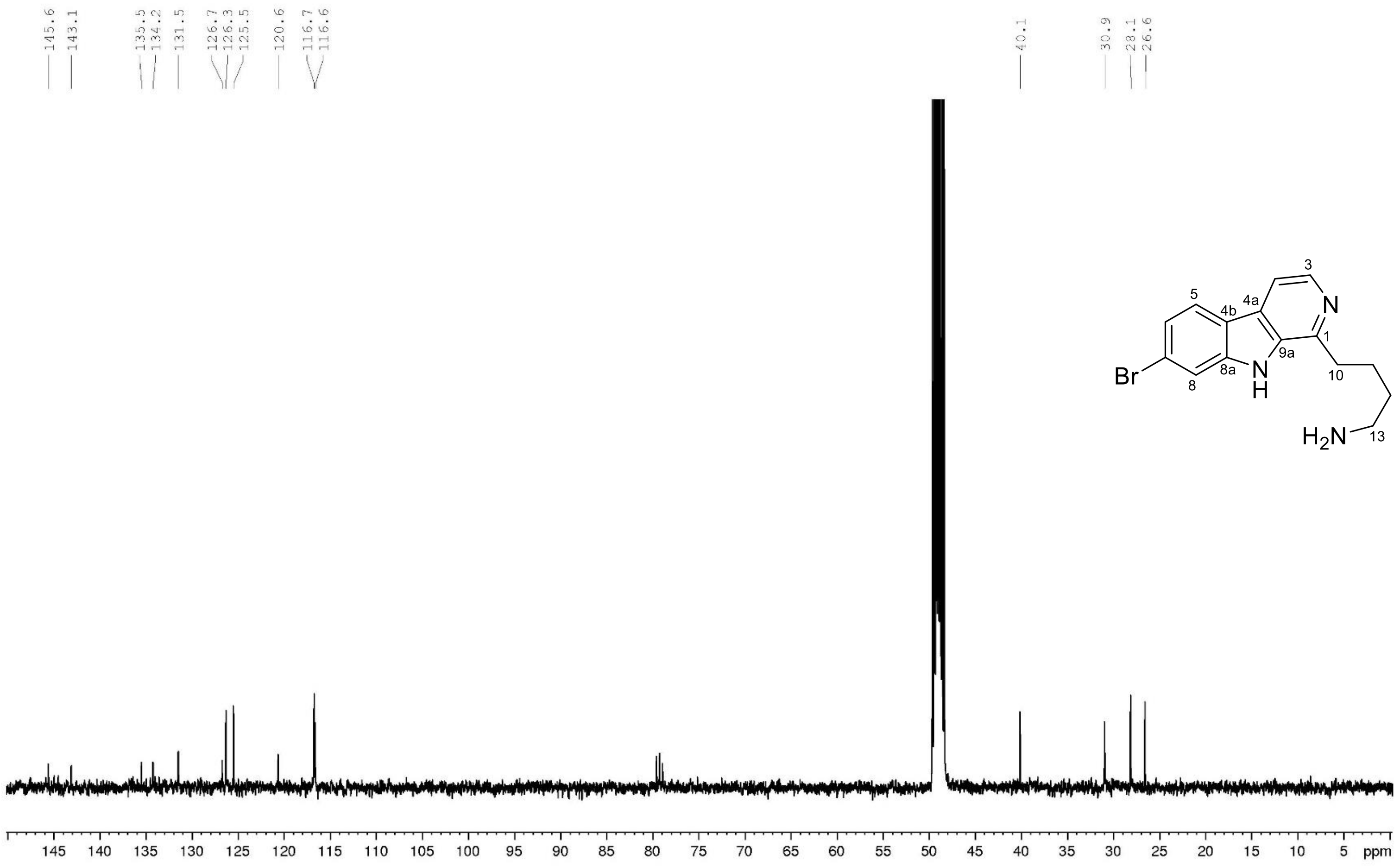

Figure S16 ^{13}C NMR spectrum of compound **14** (CD_3OD, 100 MHz).

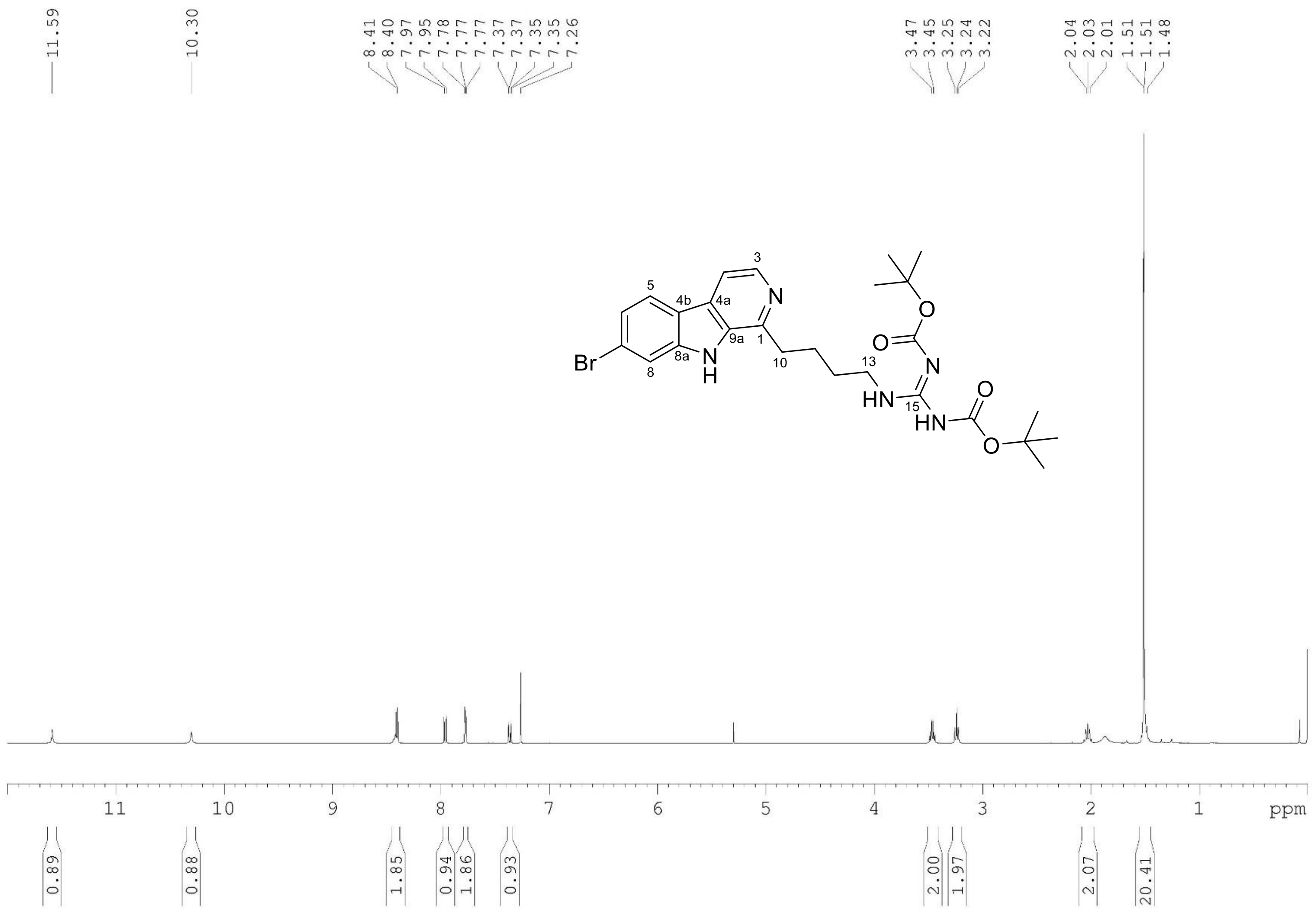

Figure S17 ^{1}H NMR spectrum of compound **16** ($CDCl_3$, 400 MHz).

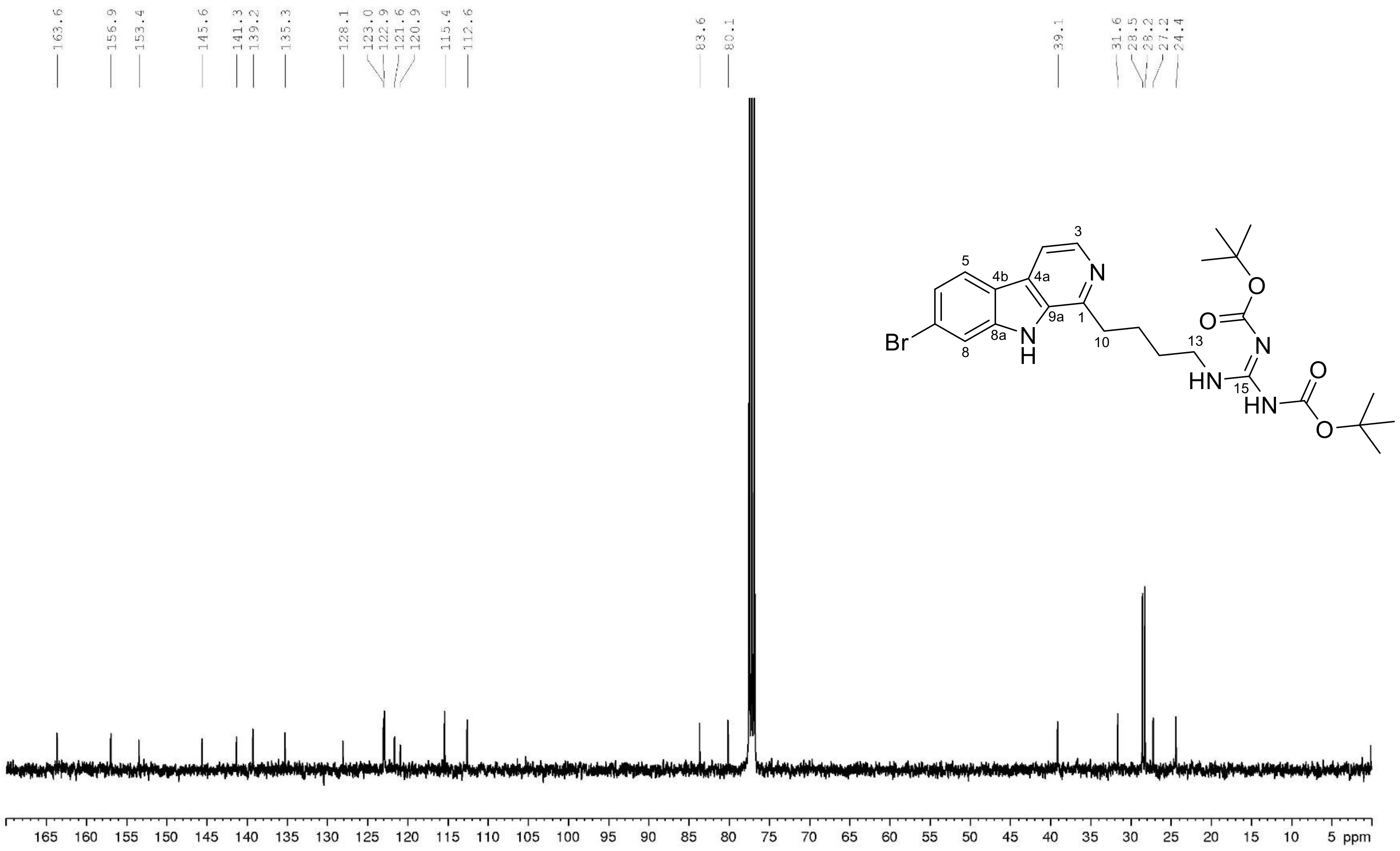

Figure S18 ^{13}C NMR spectrum of compound **16** ($CDCl_3$, 100 MHz).

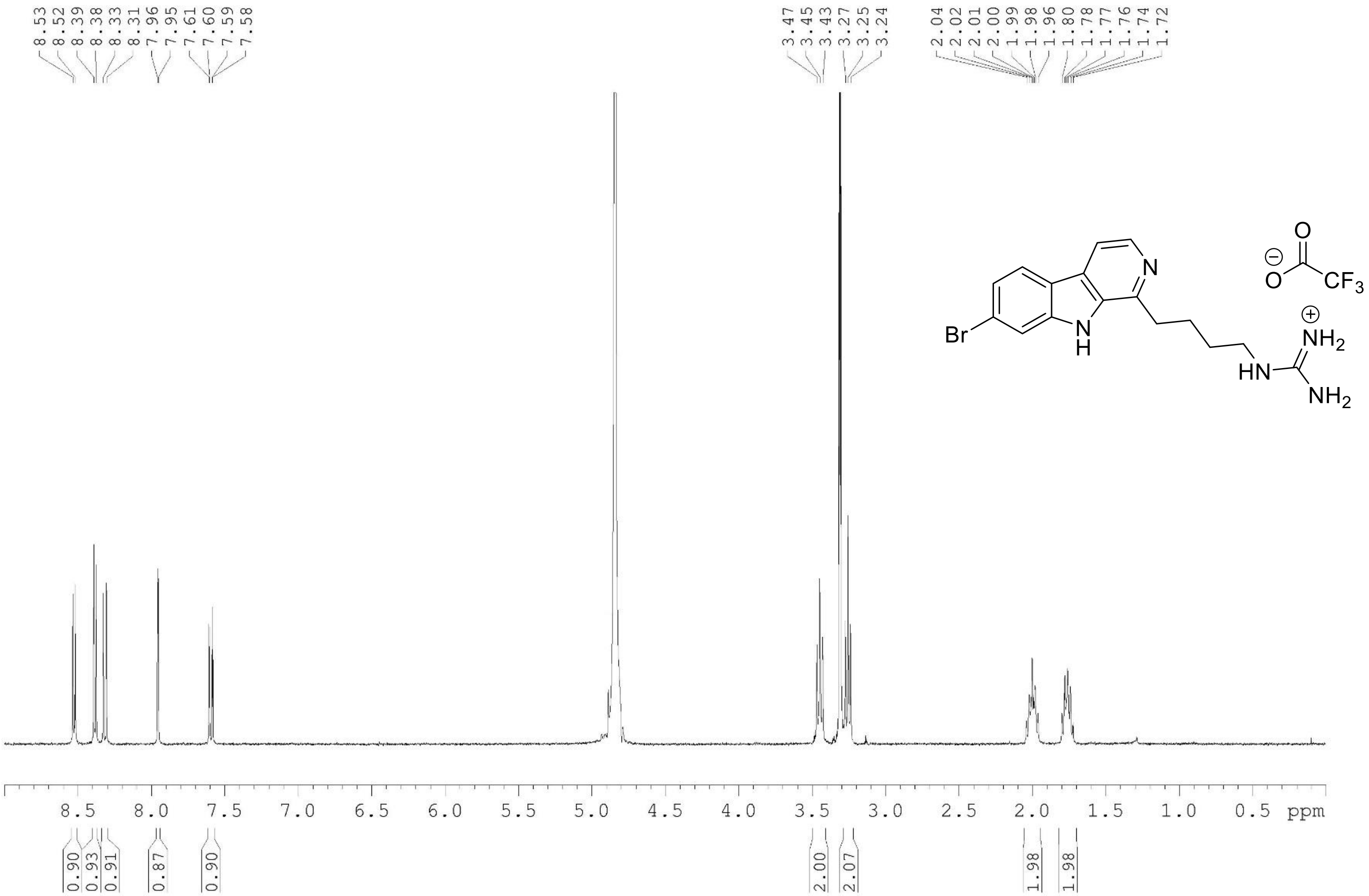

Figure S19 ^{1}H NMR spectrum of opacaline A **1** (synthetic) (CD_3OD, 400 MHz).

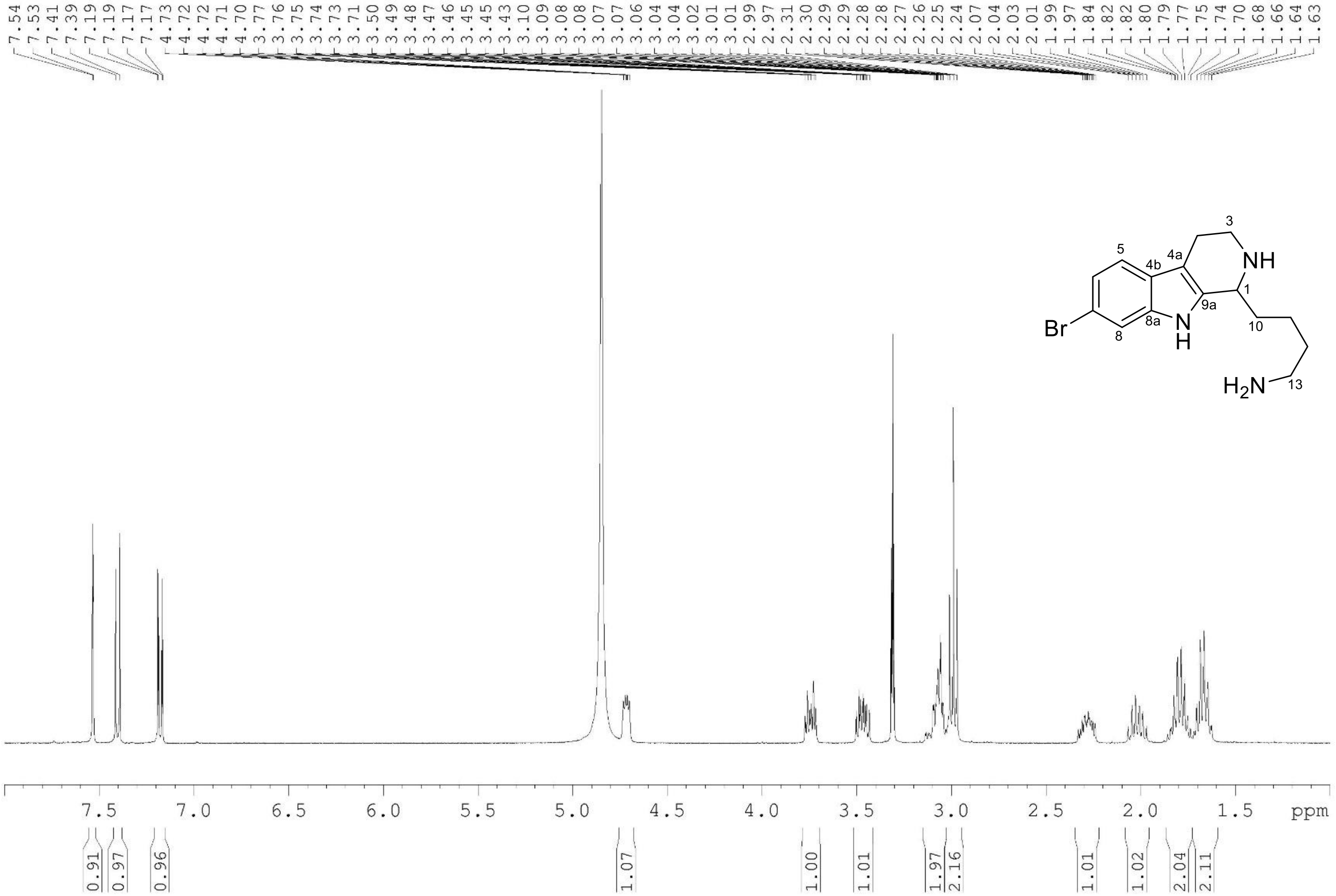

Figure S20 ^{1}H NMR spectrum of compound **18** (CD_3OD, 400 MHz).

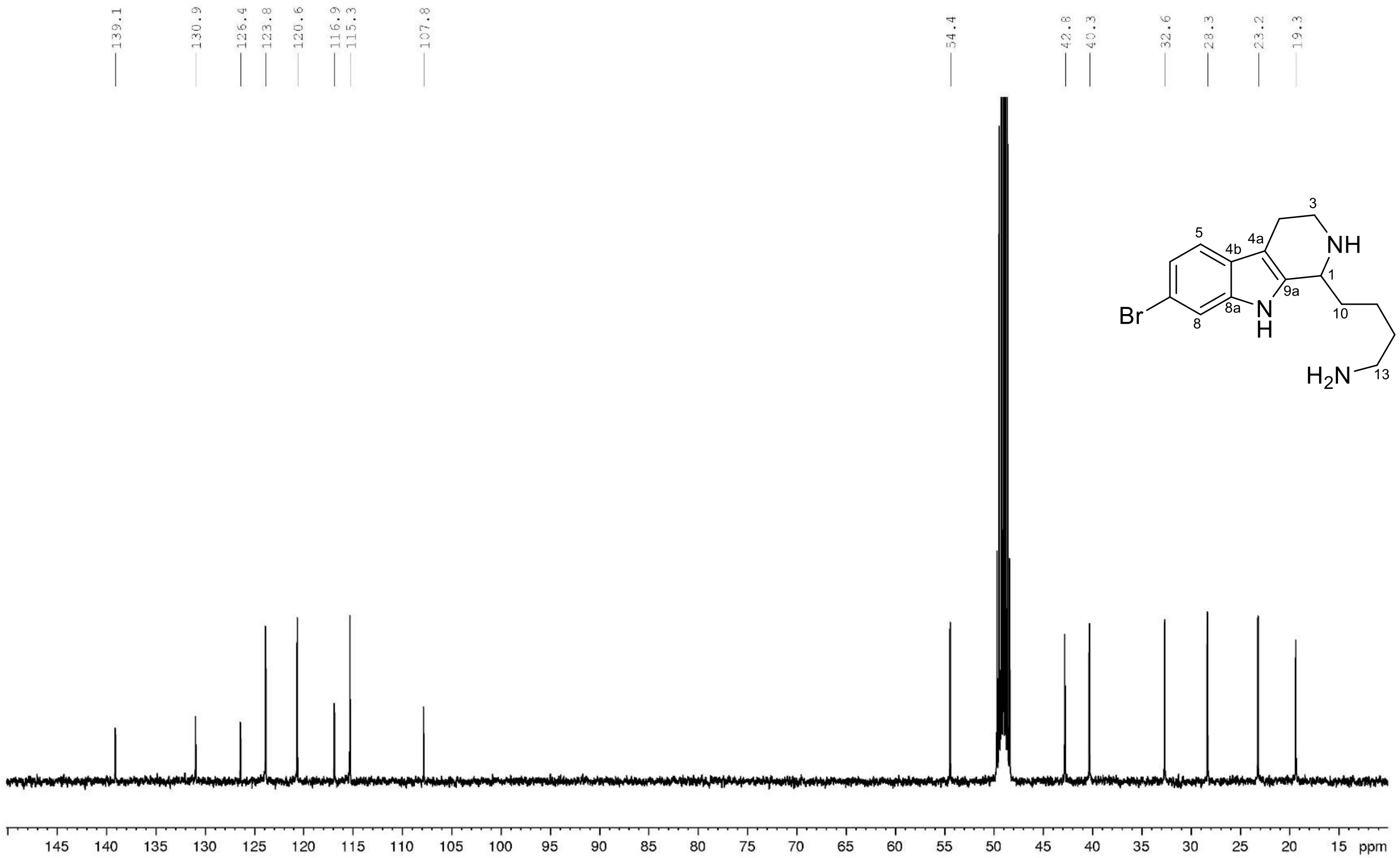

Figure S21 ^{13}C NMR spectrum of compound **18** (CD_3OD, 100 MHz).

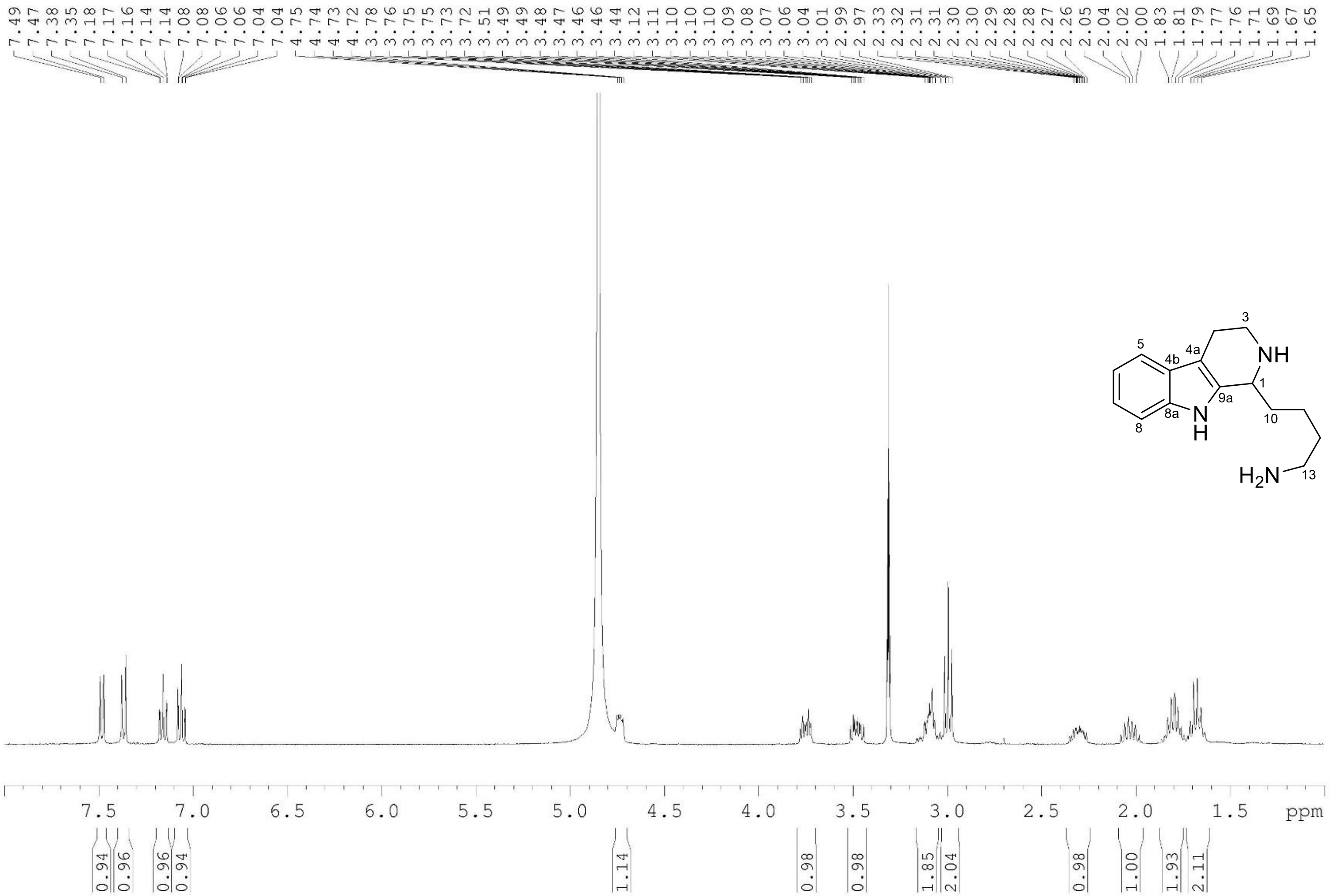

Figure S22 ^{1}H NMR spectrum of compound **19** (CD_3OD, 400 MHz).

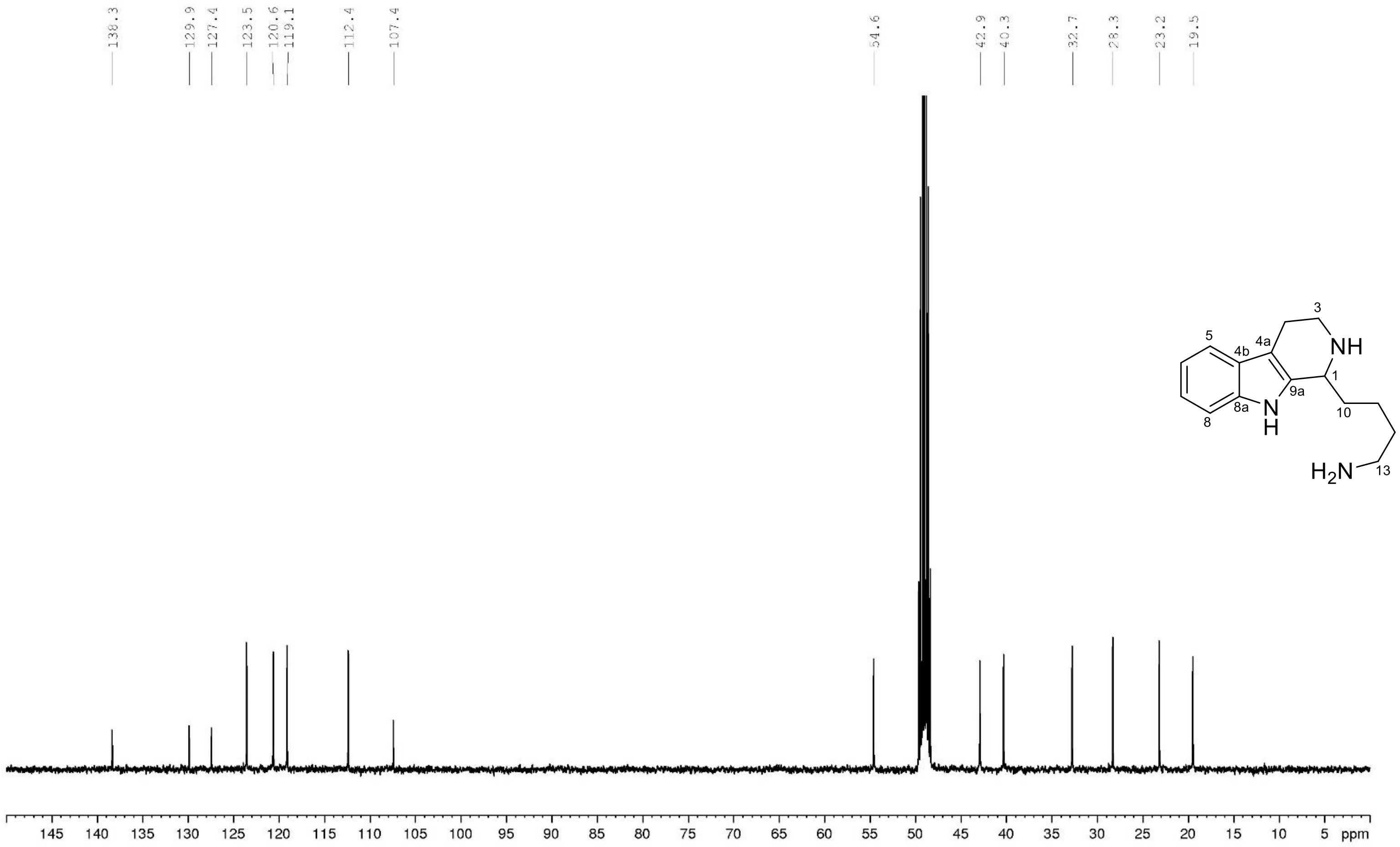

Figure S23 ^{13}C NMR spectrum of compound **19** (CD_3OD, 100 MHz).

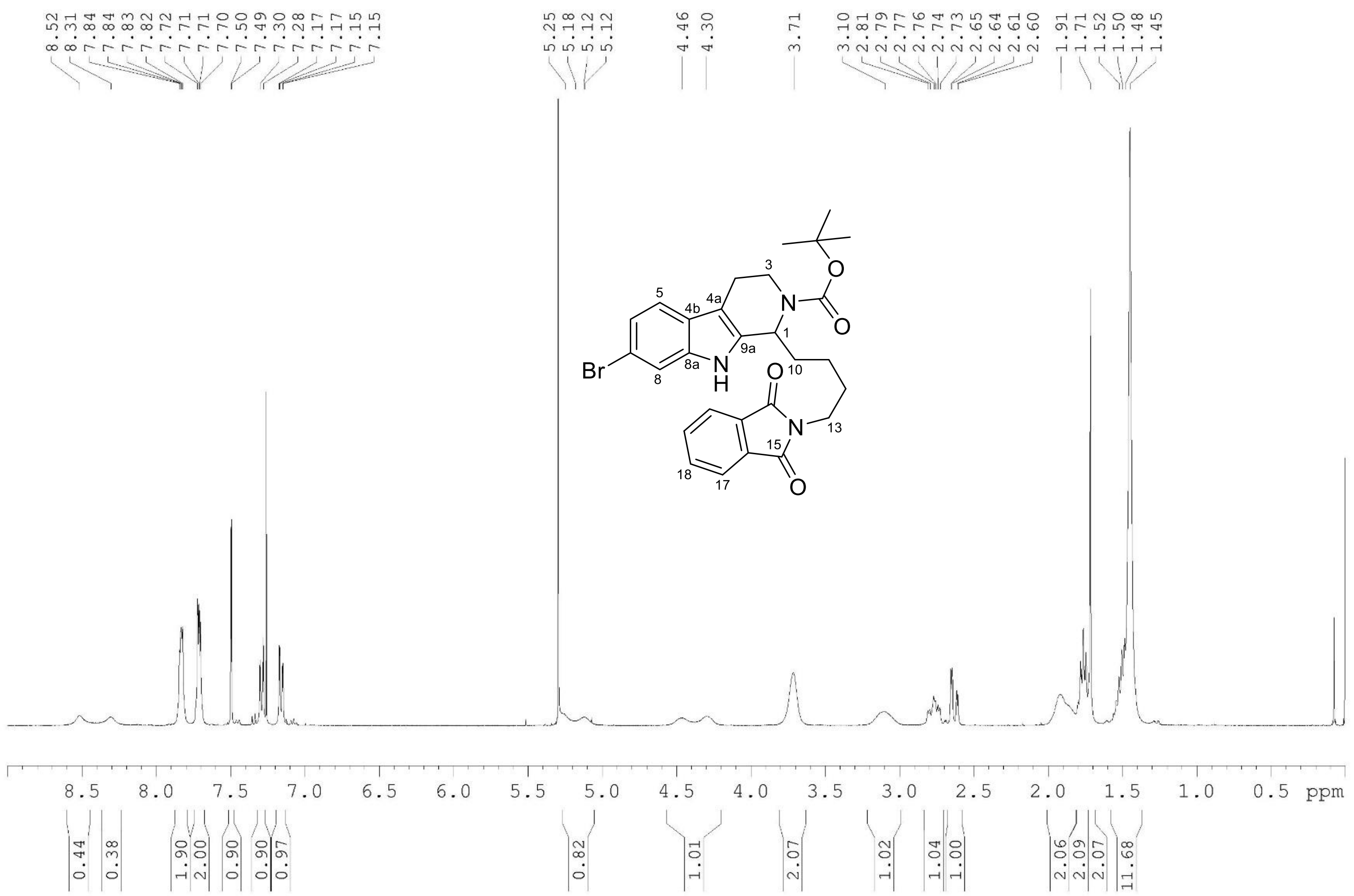

Figure S24 ^{1}H NMR spectrum of compound **20** ($CDCl_3$, 400 MHz).

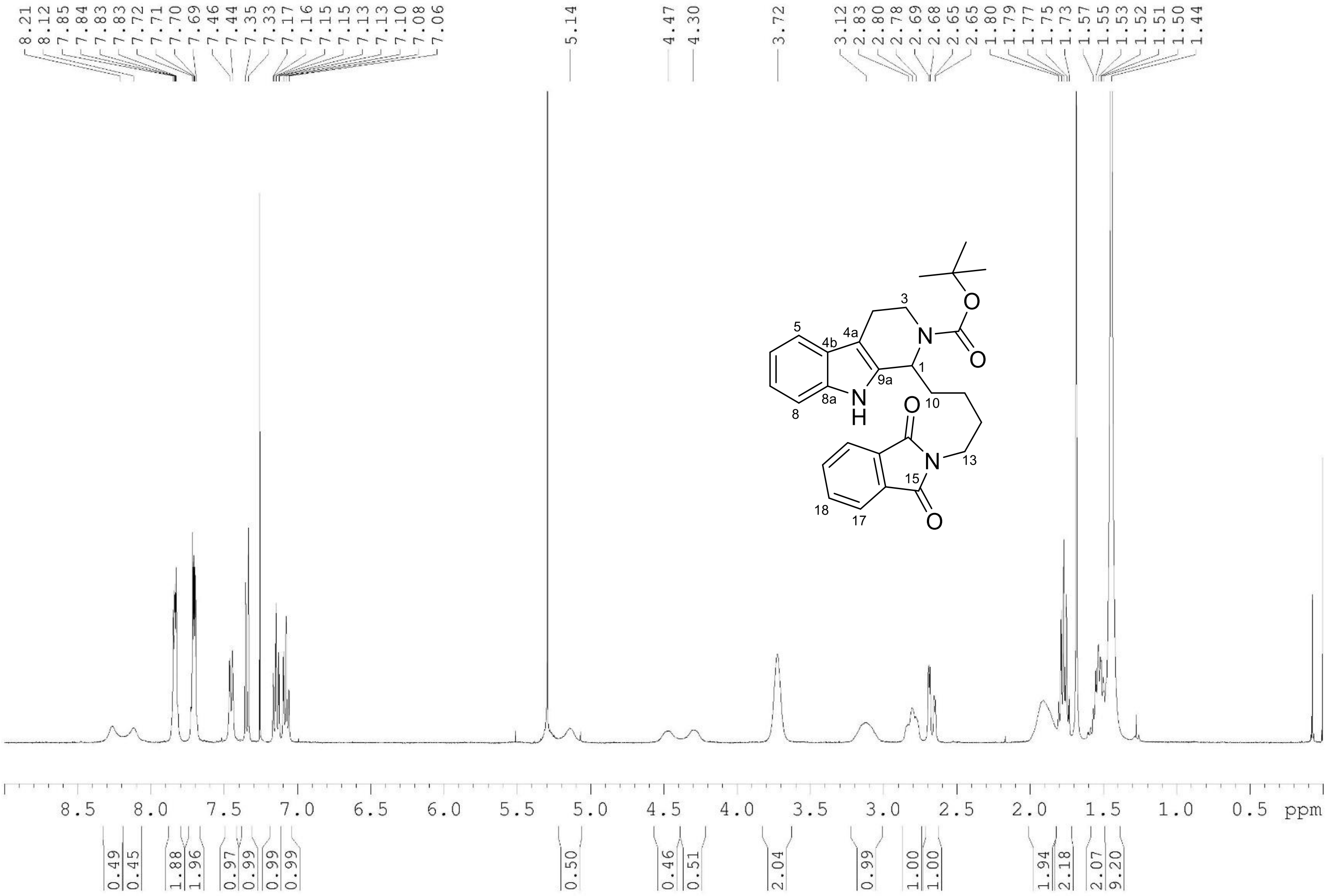

Figure S25 ^{1}H NMR spectrum of compound **21** ($CDCl_3$, 400 MHz).

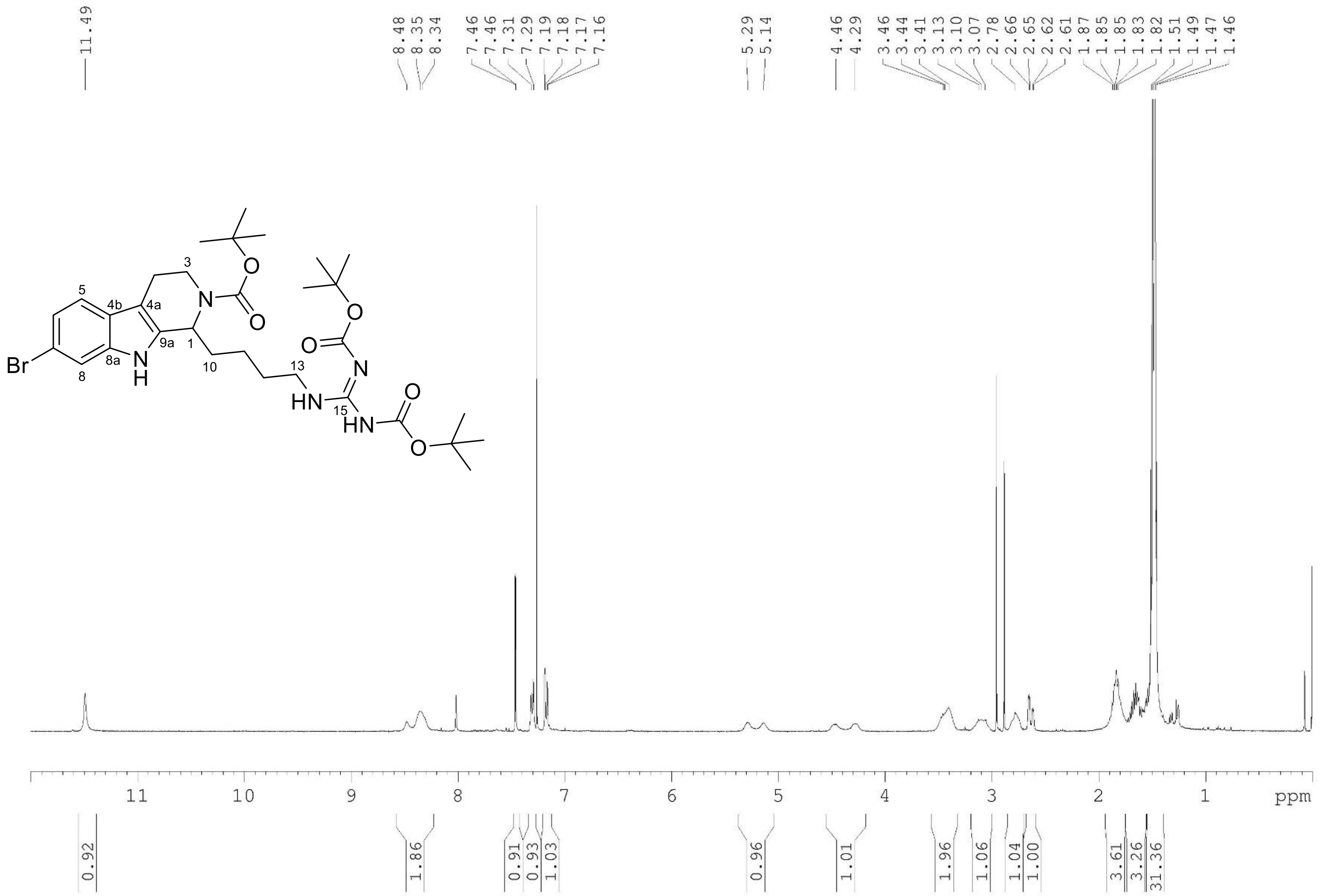

Figure S26 ^{1}H NMR spectrum of compound **22** ($CDCl_3$, 400 MHz).

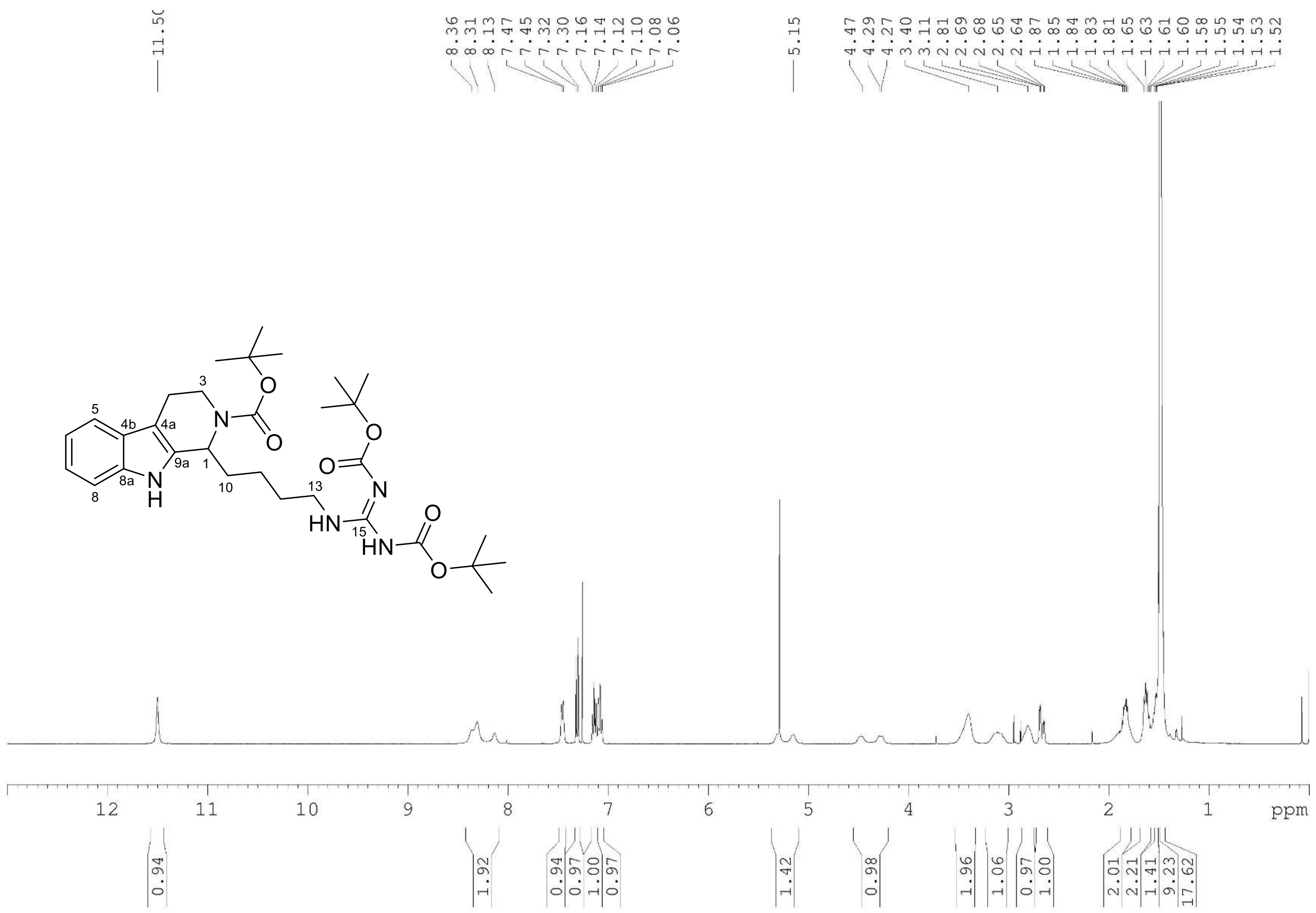

Figure S27 ^{1}H NMR spectrum of compound **23** ($CDCl_3$, 400 MHz).

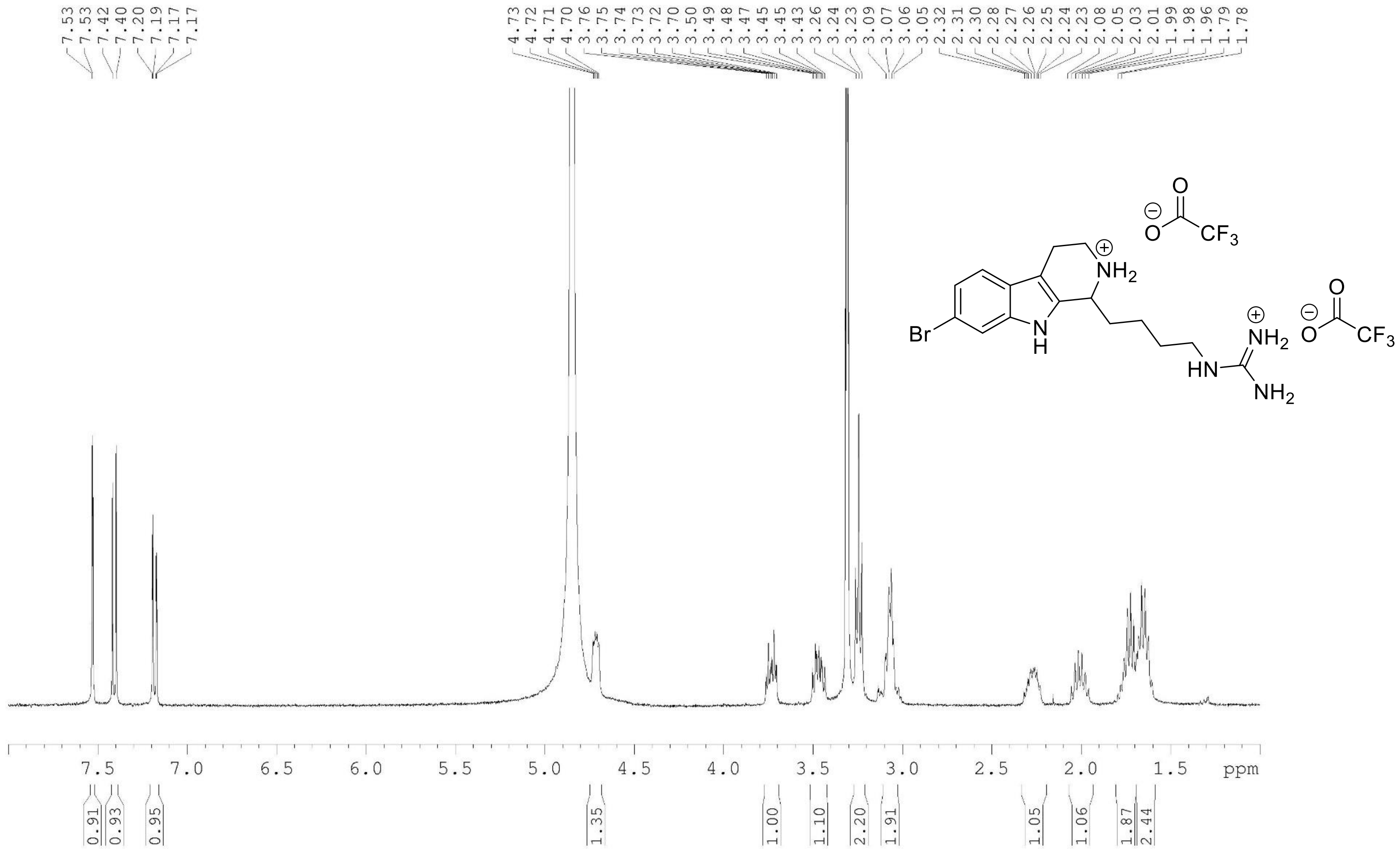

Figure S28 ^{1}H NMR spectrum of (±)7-bromohomotrypargine **3** (synthetic) (CD_3OD, 400 MHz).

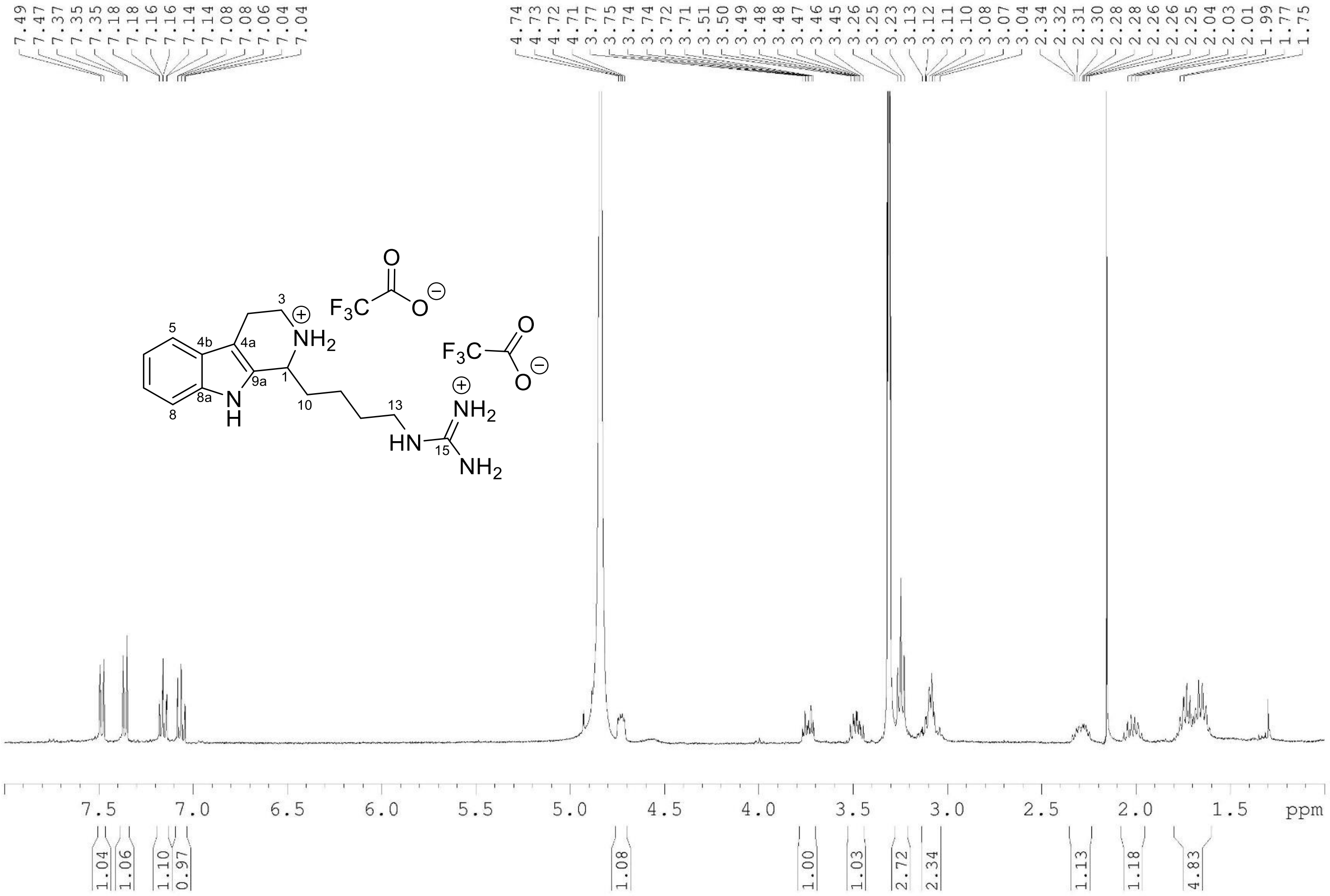

Figure S29 ^{1}H NMR spectrum of compound **24** (CD_3OD, 400 MHz).

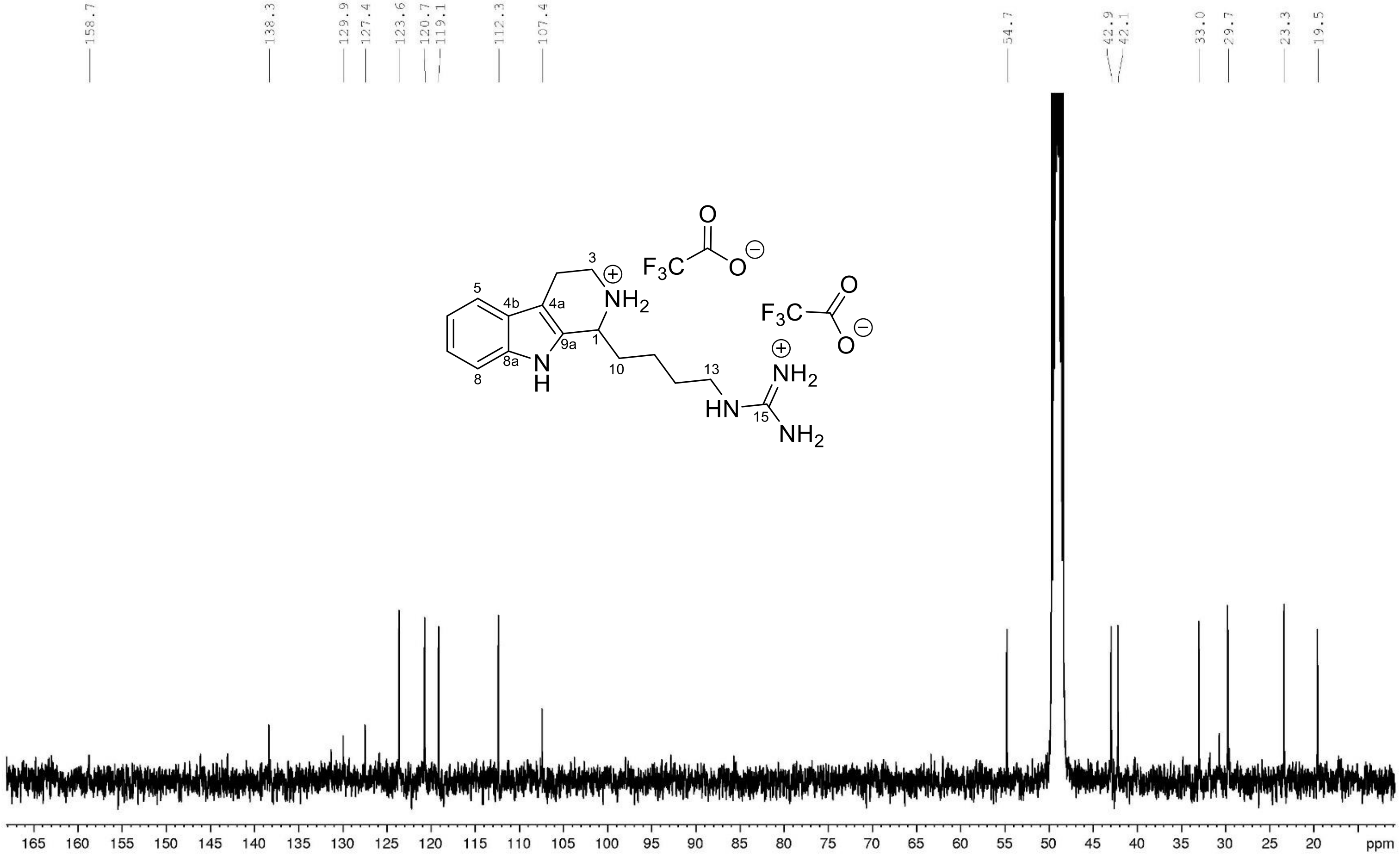

Figure S30 ^{13}C NMR spectrum of compound **24** (CD_3OD, 100 MHz).
